# Low-rate firing limit for neurons with axon, soma and dendrites driven by spatially distributed stochastic synapses

**DOI:** 10.1371/journal.pcbi.1007175

**Published:** 2020-04-20

**Authors:** Robert P. Gowers, Yulia Timofeeva, Magnus J. E. Richardson

**Affiliations:** 1 Mathematics for Real-World Systems Centre for Doctoral Training, University of Warwick, Coventry, United Kingdom; 2 Institute for Theoretical Biology, Department of Biology, Humboldt-Universität zu Berlin, Philippstrasse 13, Haus 4, Berlin, Germany; 3 Department of Computer Science, University of Warwick, Coventry, United Kingdom; 4 Department of Clinical and Experimental Epilepsy, UCL Queen Square Institute of Neurology, University College London, London, United Kingdom; 5 Warwick Mathematics Institute, University of Warwick, Coventry, United Kingdom; Inria, FRANCE

## Abstract

Analytical forms for neuronal firing rates are important theoretical tools for the analysis of network states. Since the 1960s, the majority of approaches have treated neurons as being electrically compact and therefore isopotential. These approaches have yielded considerable insight into how single-cell properties affect network activity; however, many neuronal classes, such as cortical pyramidal cells, are electrically extended objects. Calculation of the complex flow of electrical activity driven by stochastic spatio-temporal synaptic input streams in these structures has presented a significant analytical challenge. Here we demonstrate that an extension of the level-crossing method of Rice, previously used for compact cells, provides a general framework for approximating the firing rate of neurons with spatial structure. Even for simple models, the analytical approximations derived demonstrate a surprising richness including: independence of the firing rate to the electrotonic length for certain models, but with a form distinct to the point-like leaky integrate-and-fire model; a non-monotonic dependence of the firing rate on the number of dendrites receiving synaptic drive; a significant effect of the axonal and somatic load on the firing rate; and the role that the trigger position on the axon for spike initiation has on firing properties. The approach necessitates only calculating the mean and variances of the non-thresholded voltage and its rate of change in neuronal structures subject to spatio-temporal synaptic fluctuations. The combination of simplicity and generality promises a framework that can be built upon to incorporate increasing levels of biophysical detail and extend beyond the low-rate firing limit treated in this paper.

## Author summary

Neurons are extended cells with multiple branching dendrites, a cell body and an axon. In an active neuronal network, neurons receive vast numbers of incoming synaptic pulses throughout their dendrites and cell body that each exhibit significant variability in amplitude and arrival time. The resulting synaptic input causes voltage fluctuations throughout their structure that evolve in space and time. The dynamics of how these signals are integrated and how they ultimately trigger outgoing spikes have been modelled extensively since the late 1960s. However, until relatively recently the majority of the mathematical formulae describing how fluctuating synaptic drive triggers action potentials have been applicable only for small neurons with the dendritic and axonal structure ignored. This has been largely due to the mathematical complexity of including the effects of spatially distributed synaptic input. Here we show that in a physiologically relevant, low-firing-rate regime, an approximate level-crossing approach can be used to provide an estimate for the neuronal firing rate even when the dendrites and axons are included. We illustrate this approach using basic neuronal morphologies that capture the fundamentals of neuronal structure. Though the models are simple, these preliminary results show that it is possible to obtain useful formulae that capture the effects of spatially distributed synaptic drive. The generality of these results suggests they will provide a mathematical framework for future studies that might require the structure of neurons to be taken into account, such as the effect of electrical fields or multiple synaptic input streams that target distinct spatial domains of cortical pyramidal cells.

## Introduction

Due to their extended branching in both dendritic and axonal fields many classes of neurons are not electrically compact objects, in that the membrane voltage varies significantly throughout their spatial structure. A case in point are the principal, pyramidal cells of the cortex that feature a long apical dendritic trunk, oblique dendrites, apical tuft dendrites and a multitude of basal dendrites. Excitatory synapses are typically located throughout the dendritic arbour [1], while inhibitory synapses are clustered at specific regions depending on the presynaptic cell type [2]. These cells also differ morphologically not only between different layers, but also between cells in the same layer and class [3, 4]. Many cortical cells *in vivo* fire rarely and irregularly due to the stochastic and balanced nature of the synaptic drive [5, 6]. Despite the apparent irregular firing of single neurons, computational processes are understood to be distributed across the population [7, 8] with the advantage that encoding information at a low firing rate can be energy efficient [9].

The arrival of excitatory and inhibitory synaptic pulses increases or decreases the postsynaptic voltage as well as increasing the conductance locally for a short time. Together with the spatio-temporal voltage fluctuations caused by the distributed synaptic bombardment typical of *in vivo* conditions, the increase in membrane conductance affects the integrative properties of the neuron, with reductions of the effective membrane time constant, electrotonic length constant and overall input resistance of neuronal substructures [10–12].

How different classes of neurons integrate stochastic synaptic input has been a subject of intense experimental [7, 13, 14] and theoretical [15–19] focus over the last 50 years. The majority of analytical approaches have approximated the cell as electrotonically compact and focussed on the combined effects of stochastic synaptic drive and intrinsic ion currents on the patterning of the outgoing spike train. Such models usually utilize an integrate-and-fire (IF) mechanism with some variations, and have been analysed using a Fokker-Planck approach [20–23] in the limit of fast synapses. However, this approach becomes unwieldy when synaptic filtering is included (though see [24–26]). One approximate analytical methodology, applicable to the low-firing-rate limit driven by filtered synapses is the level-crossing method of Rice [27]. In this approach, which has already been applied to compact neurons [28, 29], a system without post-spike reset is considered with the rate that the threshold is crossed from below treated as a proxy for the firing rate. The upcrossing rate and firing rate for a model with an integrate-and-fire mechanism will be similar when the rate is low, such that the effect of the previous reset has faded into insignificance by the time of the next spike.

Due partly to the sparsity of the experimental data required for model constraint, but also because of the mathematical complexity involved, few analytical results mapping from distributed stochastic synaptic input to the output firing rate for neurons with dendritic structure have followed the early work of Tuckwell [30–32]. Nevertheless there is increasing interest in the integrative and firing response of spatial neuron models [33, 34], neurons subject to and generating electric fields [35–37], and the effect of axonal load and position of the action-potential initiation region [34, 38–42]. As well the simulation-based approach using multi-compartmental reconstructions, a number of recent studies have combined analytical simplifications or reductions of cables coupled to non-linear or spike generating models. These include Morris-Lecar model neurons coupled by a quasi-active dendrite for studying the synchrony of dendritic oscillators [43], and the reduction of ball-and-stick neuron models with exponential integrate-and-fire mechanisms to investigate the effect of electric fields in the presence of synaptic drive applied at a point on the dendrite [37]. Reductions of complex dendritic arbours to a few compartments have also provided valuable insight to the role of structure in filtering high-frequency input. Using constraints to intracellular recordings, an analytical treatment of a two-compartment Purkinje neuron model has provided a mechanistic explanation for the 200 Hz resonance in the firing rate response [44]. The effects of dendritic filtering have also been included in network models studying synchronization of coupled neurons [45], and the firing rates of excitatory neuronal populations in the mean-field limit [46].

At the same time, recent advances in optogenetics and multiple, parallel intracellular recordings have made experimental measurement and stimulation of *in vivo*-like input at arbitrary dendritic locations feasible [47–51]. This potential for model constraint suggests it is timely for a concerted effort to extend the analytical framework developed for compact models driven by stochastic synapses to neurons with dendrites, soma and axon in which the voltage fluctuates in both space and time.

Here we present an analytical framework for approximating the firing rate of neurons with a spatially extended structure in a physiologically relevant low-rate regime [52–54]. To illustrate the approach we applied it to simple but exemplary neuronal geometries with increasing structural features—multiple dendrites, soma and axon—and investigated how various morphological parameters including the electrotonic length, axonal radius, number of dendrites and soma size affect the firing properties.

## Materials and methods

### Derivation of the stochastic cable equation

The cable equation for the voltage *V*(*x*, *t*) in a dendrite of constant radius *a* and axial resistivity *r*_*a*_ with leak and synaptic currents has the form [55, 56]

$$c_{\text{m}}\frac{\partial V}{\partial t}=g_{L}\left(E_{L}-V\right)+g_{s}\left(x,t\right)\left(E_{s}-V\right)+\frac{a}{2r_{\text{a}}}\frac{\partial^{2}V}{\partial x^{2}}$$ (1)

where *c*_m_, *g*_*L*_ and *g*_*s*_ are the membrane capacitance, leak conductance and synaptic conductance per unit area respectively, while *E*_*L*_ and *E*_*s*_ are the equilibrium potentials for the leak and synaptic currents. The synaptic conductance over a small area of dendrite, 2*πa*Δ_*x*_, at location *x* along the dendrite increases instantaneously by an amount *γ*_*s*_ for each incident synaptic input and then decays exponentially with time constant *τ*_*s*_ as the constituent channels close

$$2\pi a\Delta_{x}\tau_{s}\frac{\partial g_{s}}{\partial t}=-2\pi a\Delta_{x}g_{s}\left(x,t\right)+\gamma_{s}\tau_{s}\sum_{\left\{t_{sk}\right\}}\delta \left(t-t_{sk}\right).$$ (2)

Here {*t*_*sk*_} denotes the set of synaptic arrival times at location *x*. Each synaptic pulse is assumed to arrive independently, where the number that arrive in a time window Δ_*t*_ is Poisson distributed with a mean *N*_*s*_ given in terms of the dendritic section area, areal density of synapses *ϱ*_s_, and mean synaptic arrival rate *r*_*s*_

$$N_{s}=2\pi a\Delta_{x}\varrho_{s}r_{s}\Delta_{t}.$$ (3)

Note that for a Poisson process the variance will also be *N*_*s*_. The approximation of synaptic arrival as Poissonian is reasonable for cases where the interspike-interval distribution is exponential [57–59] and intervals are uncorrelated. Non-Poissonian input represents an interesting topic for further study, but is outside the scope of the current study.

### Gaussian approximation for the fluctuating conductance

For a high synaptic-arrival rate we can approximate the Poissonian impulse train by a Gaussian random number with mean *N*_*s*_/Δ_*t*_ and standard deviation $\sqrt{N_{s}}/\Delta_{t}$ (this is an extension to spatio-temporal noise of the approach taken in [20]). We introduce $\psi_{k}^{i}$ as a zero-mean, unit-variance Gaussian random number that is drawn independently for each time step *i*Δ_*t*_ and each spatial position *k*Δ_*x*_. Dividing Eq (2) by the unit of membrane area allows us to write

$$\tau_{s}\frac{\partial g_{s}}{\partial t}\approx -g_{s}+\tau_{s}\gamma_{s}r_{s}\varrho_{s}+\tau_{s}\gamma_{s}\sqrt{\frac{\varrho_{s}r_{s}}{2\pi a\Delta_{x}\Delta_{t}}}\psi_{k}^{i},$$ (4)

where the right-hand side should be interpreted as having been discretized over time, with a time step Δ_*t*_. We now define the space-time white-noise process $\xi \left(x,t\right)=\psi_{k}^{i}/\sqrt{\Delta_{x}\Delta_{t}}$ that has the properties

$$\begin{array}{ccc} \langle \xi (x,t)\rangle =0 & \text{and} & \left\langle \xi \left(x,t\right)\xi \left(x^{\prime },t^{\prime }\right)\right\rangle =\delta \left(x-x^{\prime }\right)\delta \left(t-t^{\prime }\right) \end{array}$$ (5)

and also note that in the steady state 〈*g*_s_〉 = *τ*_s_ *γ*_s_ *r*_s_ *ϱ*_s_. Returning to the cable equation, we split *g*_*s*_ and *V* into mean and fluctuating components with *g*_*s*_ = 〈*g*_*s*_〉 + *g*_*sF*_ and *V* = 〈*V*〉 + *v*_*F*_ and consider that the fluctuations are relatively weak. With this assumption we can approximate 〈*v*_*F*_ *g*_*sF*_〉 ≃ 0 resulting in an additive-noise description of the voltage fluctuations [60]. This approximation encompasses parameter ranges of physiological relevance while rendering all fluctuating variables Gaussian, thereby significantly simplifying the analyses.

$$c_{\text{m}}\frac{\partial \langle V\rangle }{\partial t}=0=g\left(E-\left\langle V\right\rangle \right)+\frac{a}{2r_{\text{a}}}\frac{\partial^{2}\left\langle V\right\rangle }{\partial x^{2}}$$ (6)

with *g* = *g*_*L*_ + 〈*g*_*s*_〉 and *E* = (*g*_*L*_*E*_*L*_ + 〈*g*_*s*_〉*E*_*s*_)/*g*. It is useful to introduce the time and space constants

$$\begin{array}{ccc} \tau_{v}=\frac{c_{\text{m}}}{g} & \;\text{and}\; & \lambda =\sqrt{\frac{a}{2gr_{\text{a}}}}. \end{array}$$ (7)

For the fluctuating component we assume that the product *g*_*sF*_*v*_*F*_ is small and obtain

$$c_{\text{m}}\frac{\partial v_{F}}{\partial t}\approx -gv_{F}+g_{sF}\left(E_{s}-\left\langle V\right\rangle \right)+\frac{a}{2r_{\text{a}}}\frac{\partial^{2}v_{F}}{\partial x^{2}}.$$ (8)

Rescaling synaptic variables

$$s=\frac{g_{sF}}{g}\left(E_{s}-\left\langle V\right\rangle \right),\;\sigma_{s}=\frac{\gamma_{s}}{2g}\left(E_{s}-\left\langle V\right\rangle \right)\sqrt{\frac{\varrho_{s}r_{s}\tau_{s}}{2\pi a\lambda }}\;$$ (9)

results in the following form for the synaptic equation

$$\tau_{s}\frac{\partial s}{\partial t}=-s+2\sigma_{s}\sqrt{\lambda \tau_{s}}\;\xi \left(x,t\right).$$ (10)

where the steady-state condition d〈*V*〉/d*t* = 0 has been used. The deterministic voltage 〈*V*〉 is generally spatially varying. However, if the synaptic equilibrium potential *E*_*s*_ is far from the effective resting voltage *E* and the fluctuating voltage remains close to *E*, then it is reasonable to approximate the noise amplitude *σ*_*s*_ as being spatially uniform with *E*_*s*_ − 〈*V*〉 ≈ *E*_*s*_ − *E*. This is applicable for mostly excitatory synaptic drive where *E*_*s*_ ∼ 0mV and *E* ∼ −60mV. Letting *v* = 〈*V*〉 − *E*_*L*_ + *v*_*F*_, *μ* = *E* − *E*_*L*_, and substituting in *τ*_*v*_ and λ, we combine Eqs (6), (8) and (10) to obtain the stochastic cable equation used in the paper

$$\tau_{v}\frac{\partial v}{\partial t}=\mu -v+\lambda^{2}\frac{\partial^{2}v}{\partial x^{2}}+s.$$ (11)

Here *μ* and *s* comprise the constant and fluctuating inputs to the dendrite. These subthreshold dynamics are supplemented by the standard integrate-and-fire threshold-reset mechanism at a trigger position *x*_th_; when the voltage at *x*_th_ exceeds a threshold *v*_th_ the voltage in the entire structure is reset to voltage *v*_re_. Under *in vivo* conditions the action-potential will back-propagate throughout the neuron with complex spatio-temporal dynamics [61–63]; however, here we are considering the low-rate case in which these transient post-spike dynamics (including any bursts) will have dissipated before the next action potential is triggered.

### Boundary conditions

**Fig 1:**
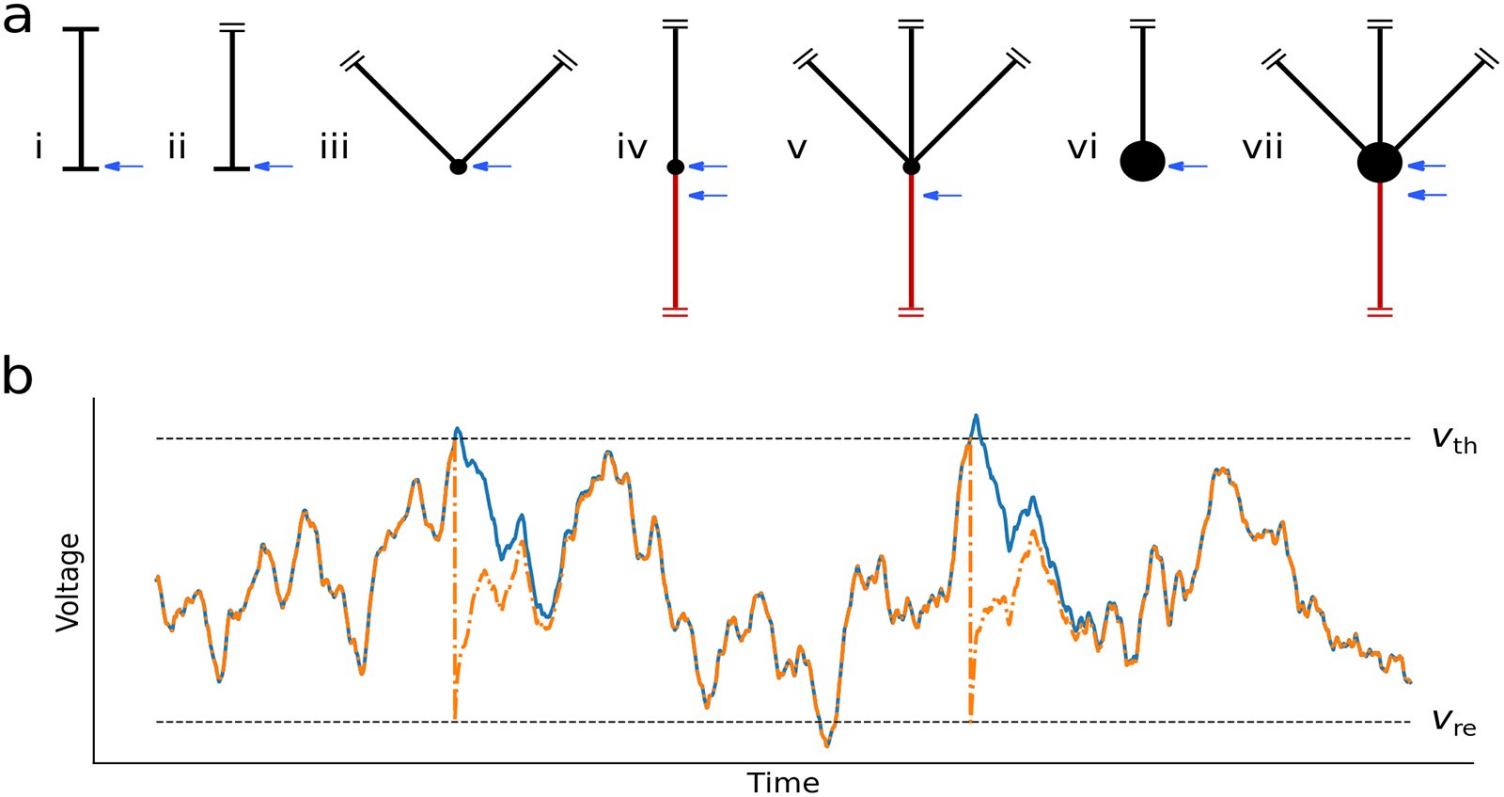
(a) The morphologies examined in this paper: (i) closed dendrite, (ii) semi-infinite one-dendrite model, (iii) two-dendrite model, (iv) dendrite and axon, (v) multiple dendrites and axon, (vi) dendrite and soma, (vii) multiple dendrites, soma and axon. Long black lines denote dendrites, red lines indicate the axon, while the blue arrows indicate the different spike trigger positions used. The other marks in the diagrams illustrate the following features: horizontal line—sealed end, two parallel lines—semi-infinite cable, small circle—nominal soma, and large circle—electrically significant soma. (b) Illustration of the upcrossing approximation. If the time between firing events is long compared to the relaxation time, the voltage without reset (solid blue line) will converge to the voltage of a threshold-reset process (orange dashed line) for the same realization of stochastic drive. Under these conditions the upcrossing and firing-rates for the two processes are comparable.

The morphologies explored in this paper are shown in Fig 1a and feature boundary conditions in which multiple dendrites and an axon meet at a soma. To account for these conditions we first define the axial current *I*_*a*_ in a cable, writing it in terms of the input conductance of an infinite cable *G*_λ_ = 2*πa*λ*g*,

$$I_{a}\left(x,t\right)=-\lambda G_{\lambda }\frac{\partial v}{\partial x}.$$ (12)

For a sealed end at *x* = 0, represented by a horizontal line in Fig 1a, no axial current flows out of the cable giving the boundary condition

$${\frac{\partial v}{\partial x}|}_{x=0}=0.$$ (13)

When the cable is unbounded and semi-infinite in extent, as shown by two small parallel lines in Fig 1a, we apply the condition that the potential must be finite at all positions,

|v(x,t)|<∞,forallx,t. (14)

For other cases, multiple (*n*) neurites join at a nominal soma *x* = 0 which is treated as having zero conductance—these cases are shown by a small circle in Fig 1a. Under these conditions the voltage is continuous at the soma *v*_1_(0) = … = *v*_*n*_(0) and axial current is conserved

$$\sum_{k=1}^{n}\lambda_{k}G_{\lambda_{k}}{\frac{\partial v_{k}}{\partial x_{k}}|}_{x_{k}=0}=0,$$ (15)

where *k* identifies the *k*th of the *n* neurites and $G_{\lambda_{k}}$ is its input conductance. Note that for each neurite the spatial variable *x*_*k*_ increases away from the point of contact *x*_*k*_ = 0. The addition of an axon changes this boundary condition by adding a cable of index *α* with length constant λ_*α*_ and conductance $G_{\lambda_{\alpha }}$. Finally, when the soma at *x* = 0 is electrically significant (denoted by a large circle in Fig 1a), there is an additional leak and capacitive current at *x* = 0. This results in a current-conservation condition

$$\tau_{0}\frac{\text{d}v_{0}}{\text{d}t}=-v_{0}+\sum_{k=\alpha ,1}^{n}\rho_{k}\lambda_{k}{\frac{\partial v_{k}}{\partial x_{k}}|}_{x_{k}=0},$$ (16)

where the subscript 0 denotes somatic quantities and the neurite dominance factor *ρ*_*k*_, which is the conductance ratio between an electrotonic length of cable and the soma $\rho_{k}\;=\;G_{\lambda_{k}}/G_{0}$ [56]. As in the case for the nominal soma, the other condition is that the voltage is continuous.

### Numerical simulation

The cable equations for each neurite with a threshold-reset mechanism were numerically simulated by implementing the Euler-Maruyama method by custom-written code in the Julia language [64]. We discretized space and time into steps Δ_*x*_ and Δ_*t*_, with *v* and *s* measured at half-integer spatial steps and the derivative ∂*v*/∂*x* at integer spatial steps. Hence, denoting *k* as the spatial index and *i* as the temporal index such that (*x*, *t*) = (*k*Δ_*x*_, *i*Δ_*t*_), $v\left(\left(k+\frac{1}{2}\right)\Delta_{x},i\Delta_{t}\right)=v_{k+1/2}^{i}$ and $\partial v/\partial v\left(k\Delta_{x},i\Delta_{t}\right)=\partial_{x}v_{k}^{i}$. The numerical algorithm used to generate *v* and *s* was therefore as follows

$$\begin{array}{ccc} v_{k+1/2}^{i+1} & = & v_{k+1/2}^{i}+\frac{\Delta_{t}}{\tau_{v}}[\mu -v_{k+1/2}^{i}+\frac{\lambda^{2}}{\Delta_{x}}\left(\partial_{x}v_{k+1}^{i}-\partial_{x}v_{k}^{i}\right)+s_{k+1/2}^{i}],\\ \partial_{x}v_{k}^{i+1} & = & \frac{v_{k+1/2}^{i+1}-v_{k-1/2}^{i+1}}{\Delta_{x}},\\ s_{k+1/2}^{i+1} & = & s_{k+1/2}^{i}+\frac{\Delta_{t}}{\tau_{s}}(-s_{k+1/2}^{i}+2\sigma_{s}\sqrt{\frac{\lambda \tau_{s}}{\Delta_{x}\Delta_{t}}}\psi_{k}^{i}), \end{array}$$ (17)

where $\psi_{k}^{i}$ denotes a zero-mean, unit-variance Gaussian random number. The code used to generate the figures is provided in the supporting information. When the approximation of an infinite or semi-infinite neurite was required, the length *L* was chosen to be sufficiently large such that boundary effects were negligible (*L* = 1000*μ*m or greater). To ensure stability of the differential equation, for a spatial step of Δ_*x*_ = 20*μ*m, we used a time step of Δ_*t*_ = 0.02 ms. We verified that this spatial step size was sufficiently small in comparison to the values of λ used by checking simulations for convergence at smaller Δ_*x*_ and Δ_*t*_.

## Results

Before examining more complex spatial models with multiple dendrites, soma and axon, we first review the subthreshold properties of a single closed dendrite driven by fluctuating, filtered synaptic drive. We then illustrate how the upcrossing method can be applied to spatial models by interpreting the results for the closed dendrite as either a long dendrite with a nominal soma at one end or as two long dendrites meeting at a nominal soma. More complex neuronal geometries are then considered including those with multiple dendrites, axon and an electrically significant soma. The parameter ranges used are given in Table 1.

**Table 1:** Parameters and their default values used in the simulations. Since many of the parameters are interdependent, where a value is not given, a formula for how it is derived from the other parameters is given instead.

Parameter	Units	Values used/Formula
*E*_*L*_	mV	-70
*E*_*s*_	mV	0
*μ*	mV	4-12
*ϵ*	-	(*E*_*L*_ − *E*_*s*_)/(*E*_*L*_ + *μ* − *E*_*s*_)
*τ*_1_	ms	10
*τ*_*α*_	ms	*ϵτ*_1_
*τ*_*s*_	ms	5
λ_1_	*μ*m	200
λ_*α*_	*μ*m	100, 150, 200
*a*_*α*_/*a*_1_	-	$g_{\alpha }\lambda_{\alpha }^{2}/\left(g_{1}\lambda_{1}^{2}\right)$
*ρ*_1_	-	1-16
*ρ*_*α*_	-	$\rho_{1}\lambda_{\alpha }^{3}/\left(\lambda_{1}^{3}\epsilon^{2}\right)$
*σ*_*s*_	mV	1-3
*v*_th_	mV	10
*v*_re_	mV	0
*x*_th_	*μ*m	0, 30

### Subthreshold properties of a closed dendrite

The dendrites considered here are driven by distributed, filtered synaptic drive. For reasons of analytical transparency, excitatory and inhibitory fluctuations are lumped into a single drive term *s*(*x*, *t*), though it is straightforward to generalize the synaptic fluctuations to two distinct processes. The fluctuating component of the synaptic drive obeys the following equation

$$\tau_{s}\frac{\partial s}{\partial t}=-s+2\sigma_{s}\sqrt{\lambda \tau_{s}}\;\xi \left(x,t\right)$$ (18)

parametrized by a filter time constant *τ*_*s*_, amplitude *σ*_*s*_ and driven by spatio-temporal Gaussian white noise *ξ*(*x*, *t*) (see Materials and methods for links to underlying presynaptic rates and density, as well as the autocovariance of *ξ*(*x*, *t*)). In anticipation of its appearance in the voltage equation, a constant λ with units of length also appears (see below). Note that the fluctuating component of the synaptic drive *s*(*x*, *t*) is a temporally filtered but spatially white Gaussian process. The subthreshold voltage in the dendrite, driven by these synaptic fluctuations, will also be a fluctuating Gaussian process and obeys the following cable equation

$$\tau_{v}\frac{\partial v}{\partial t}=\mu -v+\lambda^{2}\frac{\partial^{2}v}{\partial x^{2}}+s,$$ (19)

The time constant *τ*_*v*_ sets the duration over which a uniform perturbation of the voltage decays back to baseline whereas the space constant λ sets the length scale over which the voltage relaxes to baseline away from a point-like perturbation. Both the time and length constants are reduced by the tonic conductance increase coming from the mean component of the synaptic drive (again, see Materials and methods for derivation) and *μ* is the effective resting potential. For a closed dendrite of length *L*, shown in Fig 1a(i), there are two additional zero spatial-gradient conditions on *v*(*x*, *t*) at *x* = 0 and *x* = *L*, Eq (13). With these definitions, it is straightforward to derive 〈*v*〉, $\sigma_{v}^{2}$ and $\sigma_{\dot{v}}^{2}$ by using Green’s functions (see Eqs S22 and S15). The resulting variances can then be more succinctly written by defining the function

$$C\left(x,\eta \right)=\frac{\text{cosh}\left(\left(L-x\right)\sqrt{\eta }/\lambda \right)\text{cosh}\left(x\sqrt{\eta }/\lambda \right)}{\sqrt{\eta }\text{sinh}\left(L\sqrt{\eta }/\lambda \right)}.$$ (20)

Hence in terms of this function *C*(*x*, *η*), the variance is

$$\sigma_{v}^{2}\left(x\right)=\frac{2\sigma_{s}^{2}\tau_{s}}{\tau_{v}}\{C(x,1)-C(x,\kappa )\},$$ (21)

where *κ* = 1 + *τ*_*v*_/*τ*_*s*_. Similarly from Eq (S16), the variance of $\dot{v}$ is

$$\sigma_{\dot{v}}^{2}\left(x\right)=\frac{2\sigma_{s}^{2}}{\tau_{v}\tau_{s}}C\left(x,\kappa \right).$$ (22)

**Fig 2:**
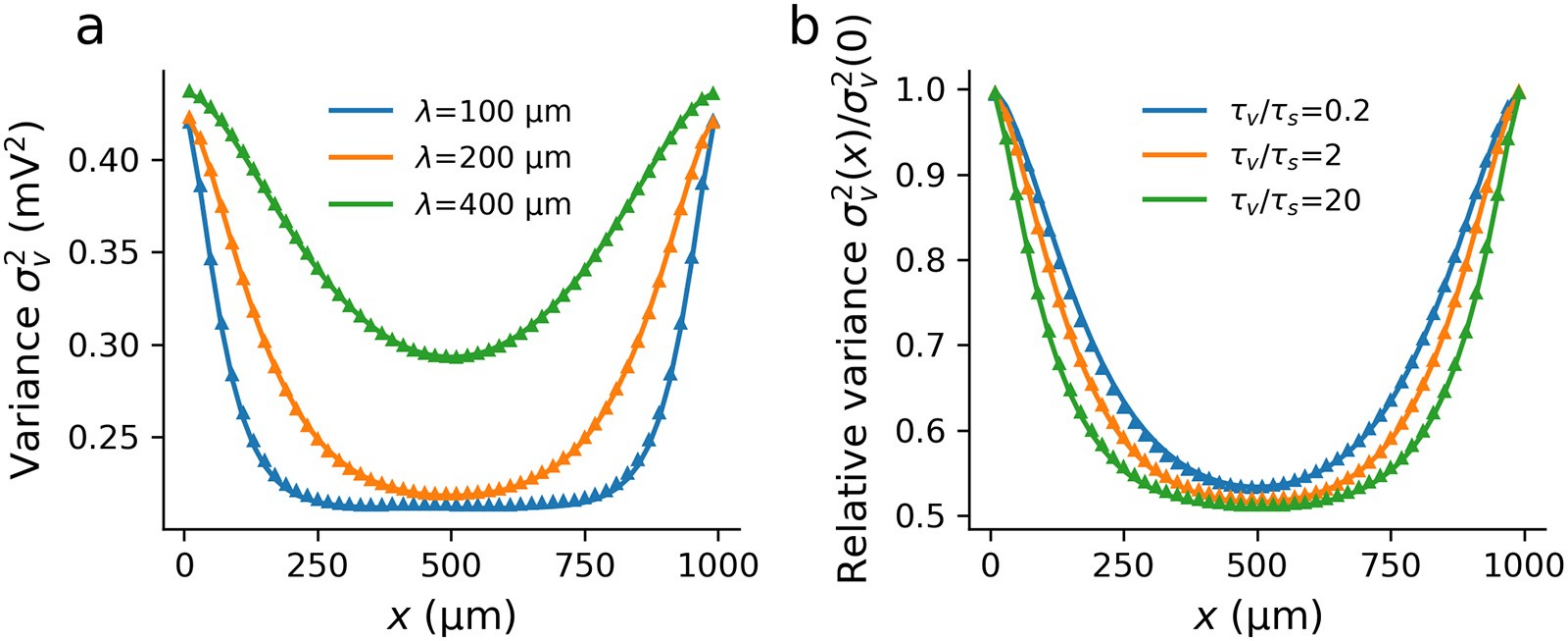
The variance profile in a sealed dendrite is a function of both the electrotonic length λ and ratio of synaptic to voltage time constants *τ*_*s*_/*τ*_*v*_. (a) The variance near the sealed end is higher than in the bulk with the extent of the boundary effect decreasing with λ. (b) Normalizing the variance in the cable such that the variance at the ends is unity, it can be seen that increasing *τ*_*v*_/*τ*_*s*_ decreases the effective length constant. For both plots the other parameters were *L* = 1000*μ*m, *τ*_*s*_ = 5ms, *σ*_*s*_ = 1mV. (a) *τ*_*v*_ = 10ms, (b) λ = 200*μ*m.

Note that the second term in the voltage-variance equation, Eq (21), and the variance of the voltage rate-of-change feature a second, shorter length constant $\lambda /\sqrt{\kappa }$ that is a function of the ratio of voltage to synaptic time constants. As expected, Fig 2a shows decreasing λ leading to a lower overall variance as well as a faster decay to the bulk properties from the boundaries. We also see from *κ* that the relative size of the time constants affects not just the magnitude of the variance but also its spatial profile. For higher *τ*_*v*_/*τ*_*s*_, $\sigma_{v}^{2}$ decreases at all positions and the profile decays faster to the bulk value as the second length constant decreases. By measuring $\sigma_{v}^{2}\left(x\right)$ relative to the variance at the ends Fig 2b shows the latter effect, though this reduction in the effective length constant by increasing *τ*_*v*_/*τ*_*s*_ is not as significant as decreasing λ.

Note that for the cases where λ/*L* ≪ 1, which is physiologically relevant for the high-conductance state, the influence of the boundary at *L* is negligible at *x* = 0 and at the midpoint there is little influence from either boundary. With this in mind, the morphologies treated in this paper comprise neurites that are treated as semi-infinite in length.

### Firing rate approximated by the upcrossing rate

Full analytical solution of the partial differential Eqs (18) and (19) when coupled to the integrate-and-fire mechanism does not appear straightforward, even for the simple closed-dendrite model. However, a level-crossing approach developed by Rice [27] and exploited in many other areas of physics and engineering, such as wireless communication channels [65], sea waves [66], superfluids [67] and grown-surface roughness [68] has previously been applied successfully to compact neuron models [28, 29] and can be extended to spatial models. The method provides an approximation for the mean first-passage time for any Gaussian process in which the mean 〈*v*〉, standard deviation *σ*_*v*_, and rate-of-change standard deviation $\sigma_{\dot{v}}$ are calculable. The upcrossing rate is the frequency at which the trajectory of *v* without a threshold-reset mechanism crosses *v*_th_ from below (i.e. with $\dot{v}\;>\;0$). Example voltage-time traces for the model with and without threshold are compared in Fig 1b. This approach provides a good approximation to the rate with reset when the firing events are rare and fluctuation driven, making it applicable to the physiological low-rate firing regime. The upcrossing rate can be derived by considering the rate at which the voltage *v*(*x*, *t*) crosses threshold *v* with a positive “velocity” $\dot{v}$ therefore

$$r_{\text{uc}}=\int_{0}^{\infty }\text{d}\dot{v}p\left(\dot{v},v\right)\dot{v}=p\left(v\right)\int_{0}^{\infty }\text{d}\dot{v}p\left(\dot{v}|v\right)\dot{v}.$$ (23)

Then using the fact that in the steady state $\dot{v}$ and *v* are independent so $p\left(\dot{v}|v\right)=p\left(\dot{v}\right)$, that $\langle \dot{v}\rangle =0$ and that both variables are considered Gaussian, we arrive at Rice’s formula [27] for the upcrossing rate across a threshold *v*_th_

$$r_{\text{uc}}=\frac{1}{2\pi }\frac{\sigma_{\dot{v}}}{\sigma_{v}}\text{exp}(-\frac{{\left(v_{\text{th}}-\left\langle v\right\rangle \right)}^{2}}{2\sigma_{v}^{2}})$$ (24)

where the statistical measures of the voltage are those at the trigger point *x*_th_. Note that because of the requirement that $\sigma_{\dot{v}}$ exists the upcrossing method cannot be applied to neurons driven by temporal white noise. However, it works well for coloured-noise drive, which is not directly tractable using standard Fokker-Planck approaches even for point-neuron models. The mean and variances required for the upcrossing Eq (24) can be found using the Green’s functions of the corresponding set of cable equations for a particular morphology and, since we only need $\left(\langle v\rangle ,\sigma_{v},\sigma_{\dot{v}}\right)$ at *x*_th_, we only need the Green’s function for the neurite that contains the trigger position (see Supporting Information for details). We now illustrate this using two interpretations of the closed-dendrite model, the one-dendrite model which focuses on the behaviour at a sealed end—Fig 1a(ii)—and the two-dendrite model which focuses on the bulk—Fig 1a(iii).

### One-dendrite and two-dendrite models

The method is now applied to a neuron with a single long dendrite and nominal soma (the trigger point *x* = 0 = *x*_th_) of negligible conductance so that the end can be considered sealed. This corresponds to a section extending from a sealed end of the closed-dendrite model considered above, in the limit that *L*/λ → ∞ (Fig 1a(ii)). The variances have already been calculated for the general case (Eqs (21) and (22)) so for *x*_th_ = 0 we have

$$\begin{array}{ccc} \;\sigma_{v}^{2}=\frac{2\sigma_{s}^{2}\tau_{s}}{\tau_{v}}\;(1-\sqrt{\frac{\tau_{s}}{\tau_{s}+\tau_{v}}}) & \text{and} & \sigma_{\dot{v}}^{2}=\frac{2\sigma_{s}^{2}}{\tau_{s}\tau_{v}}\;\sqrt{\frac{\tau_{s}}{\tau_{s}+\tau_{v}}}\;. \end{array}$$ (25)

Substitution of these variances into Eq (24) yields the upcrossing approximation to the firing rate for this geometry.

A second interpretation of the closed dendrite model is to place the trigger position in the middle *x*_th_ = *L*/2 and then, in the limit *L*/λ → ∞ consider the halves as two dendrites with statistically identical properties radiating from a nominal soma (Fig 1a(iii)), again with negligible conductance. Taking these limits of the closed dendrite Eqs (21) and (22) for this case generates variances that happen to be exactly half that of the one-dendrite case

$$\begin{array}{ccc} \sigma_{v}^{2}=\frac{\sigma_{s}^{2}\tau_{s}}{\tau_{v}}(1-\sqrt{\frac{\tau_{s}}{\tau_{s}+\tau_{v}}}\;) & \text{and} & \sigma_{\dot{v}}^{2}=\frac{\sigma_{s}^{2}}{\tau_{s}\tau_{v}}\sqrt{\frac{\tau_{s}}{\tau_{s}+\tau_{v}}}, \end{array}$$ (26)

where here we have written the functional dependence of *κ* on *τ*_*v*_ and *τ*_*s*_ explicitly. Given that the voltage at *x*_th_ is affected by activity occurring within distances a few λ down attached dendrites (see Fig 2) it might reasonably be expected that the statistical quantities and therefore the firing rate at *x*_th_ would be dependent on the electronic length quantity λ. However, for both the one and two-dendrite models considered above it is clear that there is no λ dependence for the variances. Though this is unavoidable on dimensional grounds, because in either case no other quantities carry units of length once the limit *L*/λ → ∞ has been taken, the result is nevertheless a curious one.

**Fig 3:**
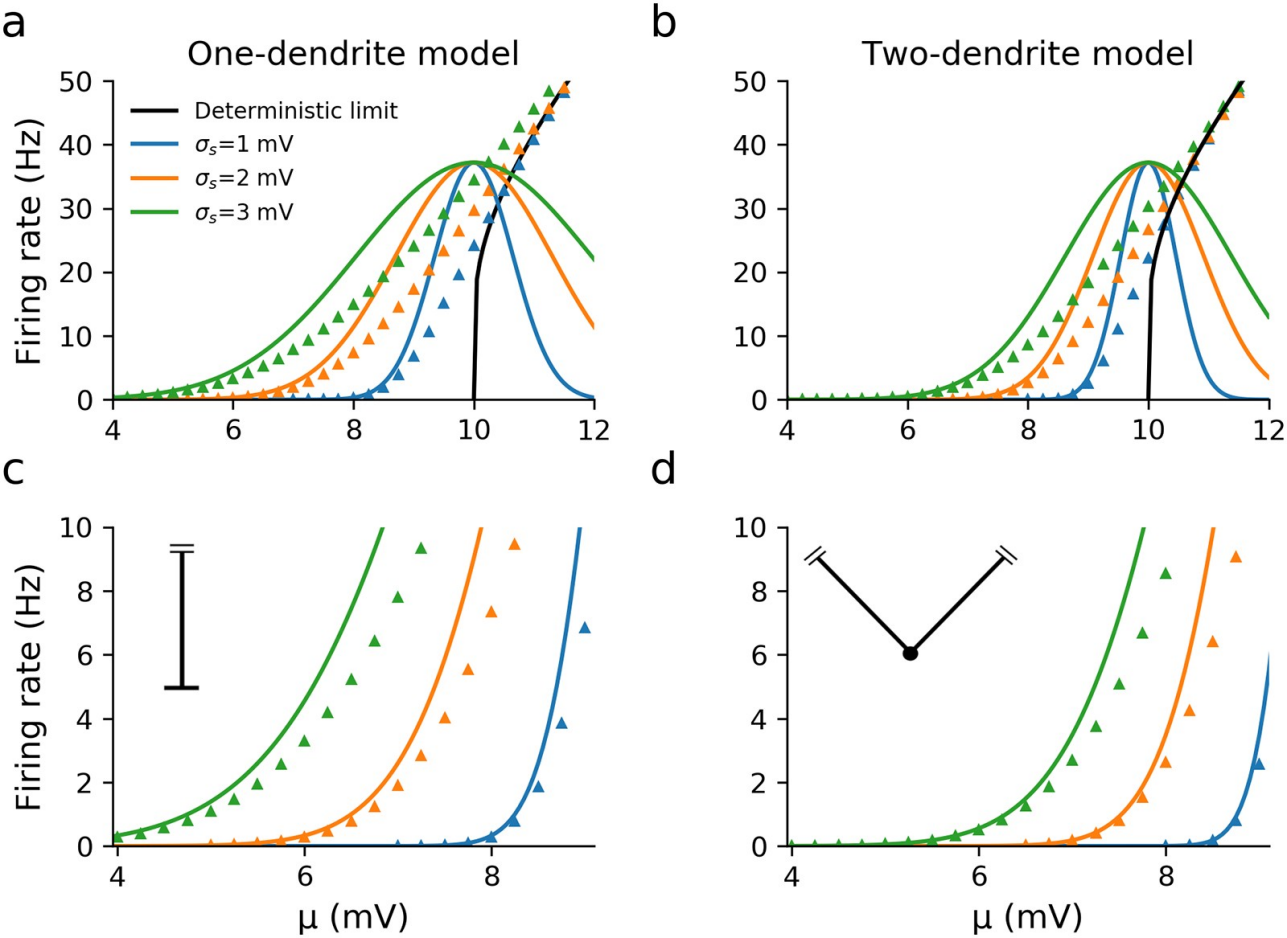
The simulated firing rates (triangles) compared with the upcrossing approximation (solid lines) of the one and two-dendrite models driven by spatially distributed, filtered stochastic synaptic drive for three fixed values of the noise amplitude *σ*_*s*_. While the firing rates of the one and two-dendrite models are similar in the suprathreshold regime (panels a and b, *μ* > 10mV), the one-dendrite model has a higher firing rate in the subthreshold regime due to the variance being twice that of the two-dendrite model for the same value of *σ*_*s*_. The upcrossing approximation is accurate when (*v*_th_ − *μ*)/*σ*_*v*_ ≫ 1 (panels c and d). Other parameters: λ = 200*μ*m, *τ*_*v*_ = 10 ms, *τ*_*s*_ = 5ms, *v*_th_ = 10mV, *v*_re_ = 0mV.

The upcrossing and firing rates as a function of *μ* for the two models are compared in Fig 3, with the deterministic firing rate also shown (this is equivalent to the deterministic rate of the leaky integrate-and-fire model). Note that we keep *τ*_*v*_ and λ constant across the range of *μ* since these parameters would change little across the range of mean synaptic drive we investigate and it allows us to isolate the dependence of the firing rate on just one parameter. The upcrossing rate provides a good approximation to the full firing rate at low rates in the < 5Hz range (see Fig A for in-depth analysis in terms of dimensionless parameters). In this way the upcrossing rate for spatio-temporal models provides a similar approximation to the firing rate as the Arrhenius form derived by Brunel and Hakim [20] for the white-noise driven point-like leaky integrate-and-fire model.

**Fig 4:**
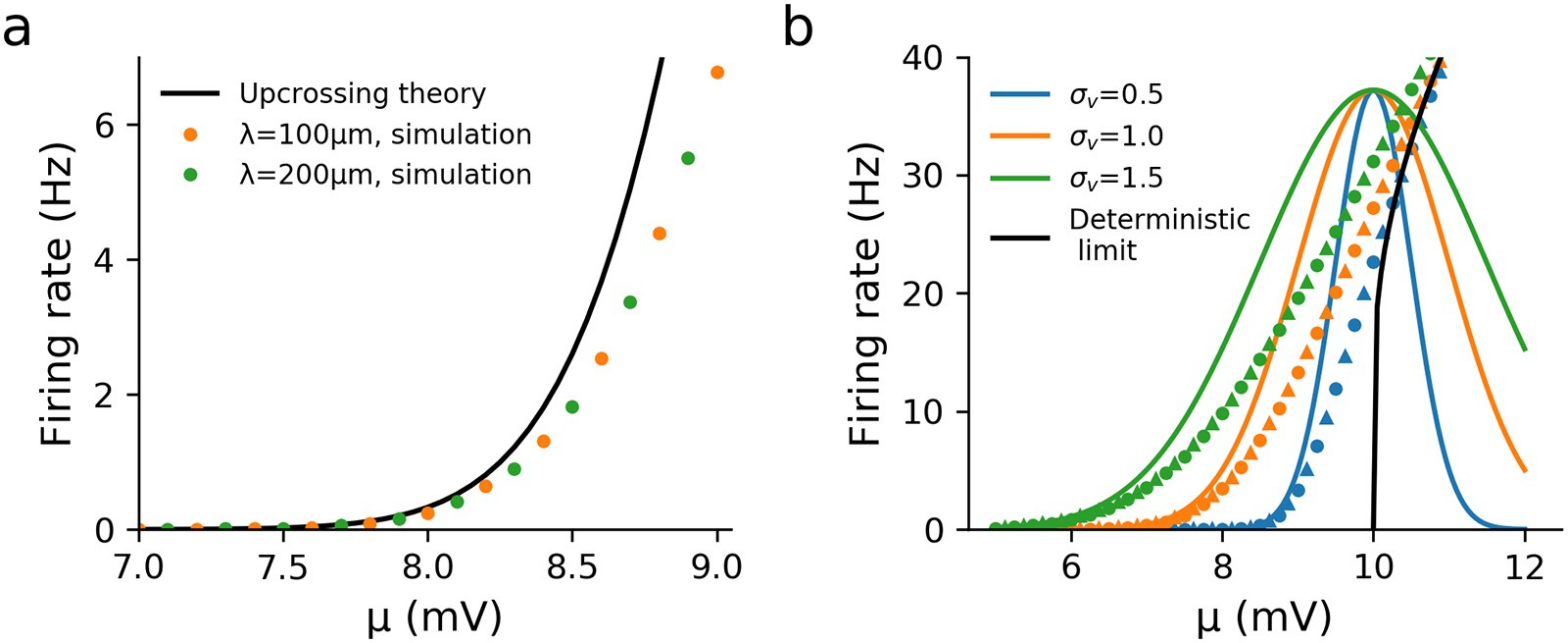
Independence of the firing rate on the electrotonic length λ for the one-dendrite model, and between the one and two-dendrite models for the same voltage variance. (a) The firing rate of the one-dendrite model with two different λ show it to be independent from λ. Here *σ*_*s*_ = 1mV. (b) If the synaptic noise amplitude *σ*_*s*_ is adjusted such that the one and two-dendrite models have the same voltage variance $\sigma_{v}^{2}$ at the threshold position, then their upcrossing rates are identical. Simulations (circles and triangles for one and two-dendrite models respectively) suggest that the full firing rates are also independent of geometry in this case. Other parameters: *τ*_*v*_ = 10ms, *τ*_*s*_ = 5ms, *v*_th_ = 10mV and *v*_re_ = 0mV.

Compared with the one-dendrite model, we see from Fig 3b and 3d that the firing rate for the two-dendrite model is significantly lower in the subthreshold regime but converges to the same value when *μ* goes above threshold. This illustrates that even simple differences in morphology affect stochastic and deterministic firing very differently. In addition Fig 4a shows that the firing rate is unaffected by the value of λ chosen, confirming by simulation the λ-independence of the firing rate. Furthermore when we choose the same value of *σ*_*v*_ for the one and two-dendrite models, then both the upcrossing rate and the simulated firing rates are the same, as seen in Fig 4b.

However, despite the independence of λ, the firing-rate profile for this toy model is distinct to that for the point-like leaky integrate-and-fire model, for which the variances are $\sigma_{v}^{2}\;\propto \;\tau_{s}/\left(\tau_{s}+\tau_{v}\right)$ and $\sigma_{\dot{v}}^{2}\;\propto \;1/\left[\tau_{s}\left(\tau_{s}+\tau_{v}\right)\right]$ [29]. This indicates that spatial structure by itself decreases the variance while increases derivative variance by a factor $\sqrt{1+\tau_{v}/\tau_{s}}$. The variances also differ in their dependence on *τ*_*v*_ and *τ*_*s*_ from two-compartmental models [69].

### Dendrite and axon

Next, we consider a dendrite connected to an axon at *x*_1_ = 0 = *x*_*α*_, as shown in Fig 1a(iv), where dendritic and axonal quantities are denoted by subscripts 1 and *α*, respectively. This differs from the previous two-dendrite model as the axon receives no synaptic drive, so *μ*_*α*_ = 0 and *s*_*α*_(*x*_*α*_, *t*) = 0. Furthermore, intrinsic membrane properties of the axon (*τ*_*α*_, λ_*α*_) differ from the dendrite due to the smaller axonal radius and lack of synapse-induced increased membrane conductance [11, 12]. Since *μ*_*α*_ = 0 we omit the subscript on the mean dendritic drive, *μ*_1_ = *μ*. Taking the reasonable assumptions that the per-area capacitance and leak conductance are the same in the axon as the soma, we can calculate *τ*_*α*_ in terms of *τ*_1_ given the mean level of synaptic drive (see Eqs S39, S41). Unlike the previous models, the mean is no longer homogeneous in space due to the lack of synaptic drive in the axon. Defining ${\tilde{f}}_{1}\left(\omega \right)$ as the input admittance of the dendrite relative to the whole neuron

$${\tilde{f}}_{1}\left(\omega \right)=\frac{G_{\lambda_{1}}\gamma_{1}}{G_{\lambda_{1}}\gamma_{1}+G_{\lambda_{\alpha }}\gamma_{\alpha }}=\frac{g_{1}^{2}\lambda_{1}^{3}\gamma_{1}}{g_{1}^{2}\lambda_{1}^{3}\gamma_{1}+g_{\alpha }^{2}\lambda_{\alpha }^{3}\gamma_{\alpha }},$$ (27)

where $\gamma_{j}=\sqrt{1+i\omega \tau_{j}}$, we can show that the mean in the axon is given by (see Eqs S13 and S19)

$$\left\langle v(x_{\alpha })\right\rangle =\mu e^{-x_{\alpha }/\lambda_{\alpha }}{\tilde{f}}_{1}\left(0\right).$$ (28)

It is important to note that, unlike in the one and two-dendrite models, Eq (28) implies that it is now possible for the neuron to still be in the subthreshold firing regime when *μ* > *v*_th_. In general, the variances do not have a closed-form solution but can be expressed in terms of the angular frequency *ω*. It can be shown that the integrand for $\sigma_{v}^{2}$ and $\sigma_{\dot{v}}^{2}$ is proportional to $|{\tilde{f}}_{1}{\left(\omega \right)|}^{2}$, Eq (S38).

**Fig 5:**
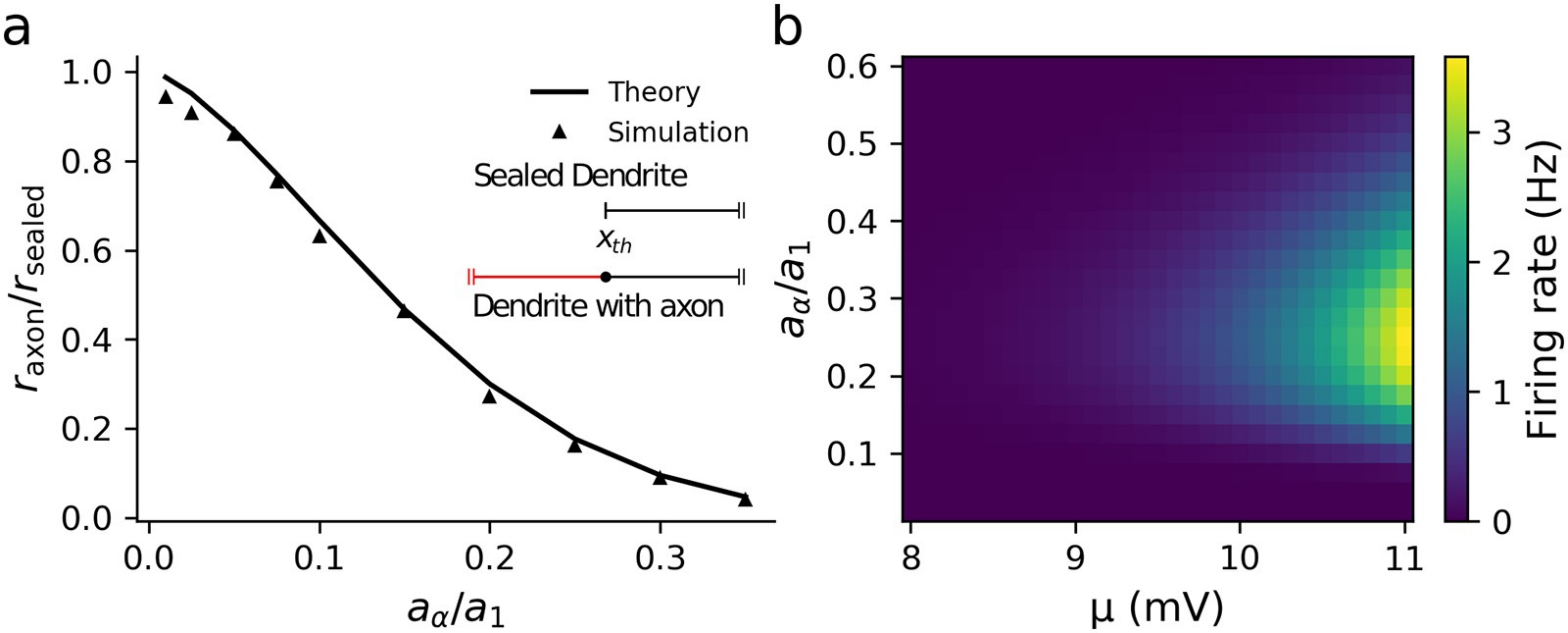
Addition of an axon significantly affects the firing rate even for small axonal conductance loads. (a) The axon increases the input conductance of the neuron, thereby lowering the firing rate for *x*_th_ = 0, *μ* = 5mV, *τ*_*α*_ = 10.8ms (calculated from Eq S39) (b) When *x*_th_ > 0 (here *x*_th_ = 30*μ*m) the firing rate varies non-monotonically with the axonal radius and peaks at a physiologically reasonable value of the ratio of axon/dendrite radii for a range of synaptic parameters. Other parameters: λ_1_ = 200*μ*m, *τ*_*s*_ = 5ms, *τ*_1_ = 10ms, *σ*_*s*_ = 3mV, *v*_th_ = 10mV and *v*_re_ = 0mV.

First we set the action-potential trigger position at *x*_th_ = 0 and evaluated the effect of the axon by comparing the firing rate for the model with an axon, *r*_axon_, to the firing rate of the one-dendrite model with a sealed end (∂*v*/∂*x* = 0) at *x* = 0, *r*_sealed_ (effectively an axon with zero conductance load). We also kept the noise amplitude *σ*_*s*_ rather than the voltage standard deviation *σ*_*v*_ fixed as we wished to see how the axon changes the variance of fluctuations at the trigger position. The relative firing rate was defined as *r*_axon_/*r*_sealed_. The ratio of the axonal to dendritic radius *a*_*α*_/*a*_1_ was varied and the relative firing rate calculated, with *a*_*α*_/*a*_1_ = 0 being equivalent to no axon present. As illustrated in Fig 5a, the addition of a very low conductance or relatively thin axon significantly reduces the firing rate. This effect arises because the magnitude of ${\tilde{f}}_{1}\left(\omega \right)$ decreases at all frequencies for a larger radius ratio, which can be understood by recalling that $\lambda_{j}\propto \sqrt{a_{j}}$, Eq (7).

For cortical pyramidal cells, action potentials are typically triggered around *x*_th_ = 30*μ*m down the axon in the axon initial segment [70–72]. It is straightforward to investigate the effect of moving the trigger position down the axon using the upcrossing approach. Interestingly, when *x*_th_ > 0, a non-monotonic relationship between the firing rate and radius ratio *a*_*α*_/*a*_1_ became apparent (see Fig 5b), with the peak ratio of ∼0.25 being similar to that between the axonal initial segment and apical dendrite diameter in pyramidal cells [41, 73]. This is caused by a non-monotonic dependence of both 〈*v*〉 and $\sigma_{v}^{2}$ on *a*_*α*_/*a*_1_ for *x*_th_ > 0 with each peaking at intermediate values. Intuitively, this can be understood from the definition of λ_*α*_, which increases as $\sqrt{a_{\alpha }}$. Thus the decay length of voltage fluctuations that enter the axon from the dendrite increases, increasing both 〈*v*〉 and $\sigma_{v}^{2}$ at *x*_th_. On the other hand, a larger λ_*α*_ increases the input conductance of the neuron, which, conversely, decreases 〈*v*〉 and $\sigma_{v}^{2}$. For smaller λ_*α*_ the decay length effect is more significant, whereas for larger λ_*α*_ the increase in input conductance plays a larger role.

### Multiple dendrites and axon

We now consider a case with multiple dendrites and an axon radiating from a nominal soma (Fig 1a(v)). The dendrites are labelled 1, 2, …, *n* with the axon labelled *α* as before. The dendrites have identical properties with independent and equally distributed synaptic drive. As in the previous case with the dendrite and axon, we kept the synaptic strength *σ*_*s*_ fixed as we changed the number of dendrites. An immediate consequence of multiple dendrites is that, since *μ* > 0 the mean voltage in the axon increases as more dendrites are added, with each contribution summing linearly,

$$\left\langle v_{\alpha }\left(x_{\alpha }\right)\right\rangle =\sum_{k=1}^{n}\left\langle v_{\alpha k}\left(x_{\alpha }\right)\right\rangle ,$$ (29)

where 〈*v*_*αk*_(*x*_*α*_)〉 is the contribution to the axonal voltage mean from dendrite *k*. Introducing the relative input admittance of a single dendrite ${\tilde{f}}_{n}\left(\omega \right)$

$${\tilde{f}}_{n}\left(\omega \right)=\frac{G_{\lambda_{1}}\gamma_{1}}{nG_{\lambda_{1}}\gamma_{1}+G_{\lambda_{\alpha }}\gamma_{\alpha }}=\frac{g_{1}^{2}\lambda_{1}^{3}\gamma_{1}}{ng_{1}^{2}\lambda_{1}^{3}\gamma_{1}+g_{\alpha }^{2}\lambda_{\alpha }^{3}\gamma_{\alpha }},$$ (30)

it can be shown that when all dendrites have identical mean input drive *μ*, the mean in the axon is given by (see Eqs S13, S25)

$$\left\langle v(x_{\alpha })\right\rangle =n\mu e^{-x_{\alpha }/\lambda_{\alpha }}{\tilde{f}}_{n}\left(0\right).$$ (31)

Thus we can see that as *n* increases the mean increases towards the constant value of $\mu e^{-x_{\alpha }/\lambda_{\alpha }}$. However, this is not the case for the fluctuating component: despite more sources of fluctuating synaptic input both $\sigma_{v}^{2}$ and $\sigma_{\dot{v}}^{2}$ in the axon decrease as 1/*n* for a large number of dendrites. We can see this by noting that for large *n*, $|{\tilde{f}}_{n}{\left(\omega \right)|}^{2}$ and hence the variance contribution from each dendrite scales as 1/*n*^2^. Therefore for *n* total dendrites, the total variance at *x*_th_ in the axon will scale as 1/*n* for large *n*. This reduction in axonal variance with additional dendrites is a generalization of the reduction in variance we saw between the one and two-dendrite models earlier in Eqs (25) and (26).

**Fig 6:**
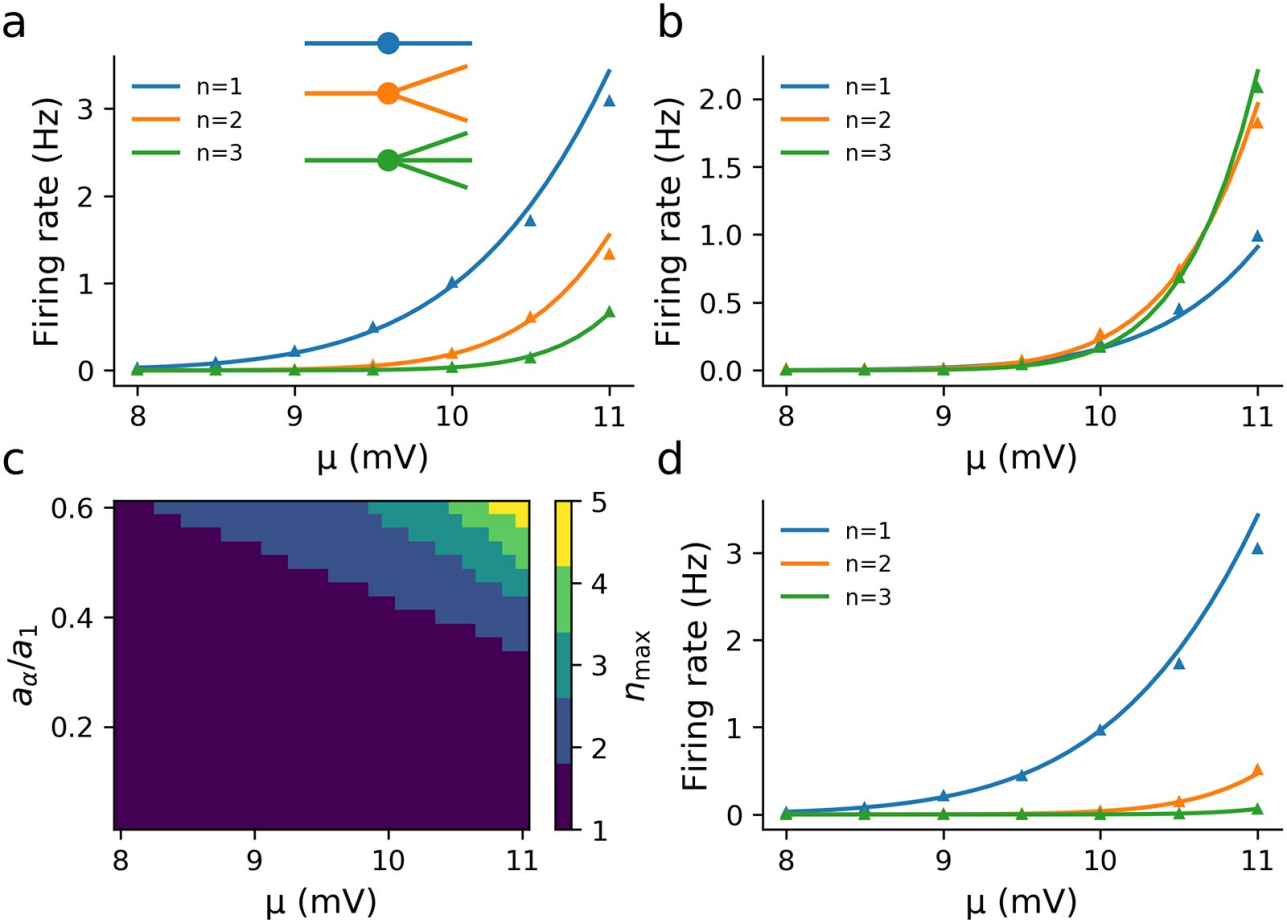
Increasing the number of synaptically driven dendrites can decrease the firing rate when the axon, of radius *a*_*α*_ is much thinner than the dendrite, radius *a*_1_. The length constant for each neurite is proportional to the square root of the radius $\lambda_{j}\propto \sqrt{a_{j}}$. (a) λ_*α*_ = 100*μ*m, (b) λ_*α*_ = 150*μ*m, (c) The number *n*_max_ of dendrites that maximizes firing increases with higher ratios of axon-to-dendrite radii *a*_*α*_/*a*_1_ and *μ*, (d) λ_*α*_ = 100*μ*m, λ_1_ = 200*μ*m for *n* = 1 and λ_1_ is rescaled for larger *n* to keep the input conductance equal to the *n* = 1 case. Other parameters: λ_1_ = 200*μ*m (a-c, kept constant with *μ* by adjusting *a*_1_, Eq S42, *a*_1_(*μ* = 11)/*a*_1_(*μ* = 8) ≈ 1.05), *τ*_1_ = 10ms, *τ*_*α*_ = 11.3—11.9ms (calculated from Eq S39), *σ*_*s*_ = 3mV, *v*_th_ = 10mV and *x*_th_ = 30*μ*m down the axon.

When it is the fluctuations that contribute significantly for firing (i.e. smaller *μ* or λ_*α*_) then a reduction in variance from adding more dendrites will decrease the firing rate; however, when the mean is more significant (larger *μ* or λ_*α*_) then the firing rate will increase as the number of dendrites increases. An example of the former case is shown in Fig 6a for λ_*α*_ = 100*μ*m, while an example of the latter is seen in Fig 6b for λ_*α*_ = 150*μ*m. The transition between these regimes can be seen in Fig 6c, which shows how the value of *n* that maximizes the firing rate, *n*_max_, increases with *μ* and *a*_*α*_/*a*_1_. Physiologically, the reduction in variance is not simply the fact that adding dendrites increases cell size and thus input conductance, but that the relative conductance of each input dendrite to the total conductance decreases. Given that the total input conductance for *n* dendrites and an axon is

$$G_{\text{in}}\left(n\right)=n\left(2\pi a_{1}\lambda_{1}g_{1}\right)+2\pi a_{\alpha }\lambda_{\alpha }g_{\alpha },$$ (32)

we can test this idea by scaling λ_1_, *a*_1_ with *n* (i.e. making the dendrites thinner) to keep the total input conductance the same as the single dendrite case, *G*_in_(*n* = 1). This gives the simple relationship λ_1_(*n*) = λ_1_(*n* = 1)/*n*^1/3^, which when substituted into the segment factor yields

$${\tilde{f}}_{n}\left(\omega \right)=\frac{\frac{1}{n}g_{1}^{2}\lambda_{1}^{3}\left(n=1\right)\gamma_{1}}{g_{1}^{2}\lambda_{1}^{3}\left(n=1\right)\gamma_{1}+g_{\alpha }^{2}\lambda_{\alpha }^{3}\gamma_{\alpha }}.$$ (33)

Since the integrands for the variances are proportional to $|{\tilde{f}}_{n}{\left(\omega \right)|}^{2}$, Eq (S38), this shows that $\sigma_{v}^{2}$ and hence the firing rate for fixed λ_*α*_ still decreases as *n* increases (see Fig 6d).

Finally, we also notice better agreement between the upcrossing rate and the simulated firing rate than the infinite dendrite case for the same output firing rates. Intuitively, this is due to the additional filtering from the spatial distance between the dendrite and trigger position along the axon.

### Dendrites, soma and axon

We now consider the case illustrated in Fig 1a(vii), where the electrical properties of the soma are non-negligible with its lumped capacitance and conductance providing an additional complex impedance at the point where the axon and dendrites meet. This has the somatic boundary condition we gave earlier in Eq (16) and we recall that the subscript 0 denotes somatic quantities. For simplicity, and as neither section receives synaptic drive in our model, we will let the somatic time constant be the same as the axonal time constant, so *τ*_0_ = *τ*_*α*_. Note that somatic drive can be straightforwardly added in this framework, as the variance contribution from the resultant fluctuations would add linearly. This would not qualitatively change the nature of the results we present here that focus on the effects of somatic filtering on transfer of dendritic stimulation to the trigger point in the axon. As the ratio of dendritic to somatic input conductance (*ρ*_1_, see Materials and methods) tends to infinity, the model without somatic drive converges to the dendrite and axon model with a nominal soma, allowing a clearer comparison between the two models.

For an electrically significant soma the integrand for the variance has the same form as before, Eq (S38), but $\tilde{f}$ now depends on the neurite dominance factor *ρ*,

$$\;{\tilde{f}}_{n0}\left(\omega \right)=\frac{G_{\lambda_{1}}\gamma_{1}}{G_{0}\gamma_{0}^{2}+nG_{\lambda_{1}}\gamma_{1}+G_{\lambda_{\alpha }}\gamma_{\alpha }}=\frac{\rho_{1}\gamma_{1}}{\gamma_{0}^{2}+n\rho_{1}\gamma_{1}+\rho_{\alpha }\gamma_{\alpha }},\;\gamma_{0}^{2}=1+i\omega \tau_{0}.$$ (34)

**Fig 7:**
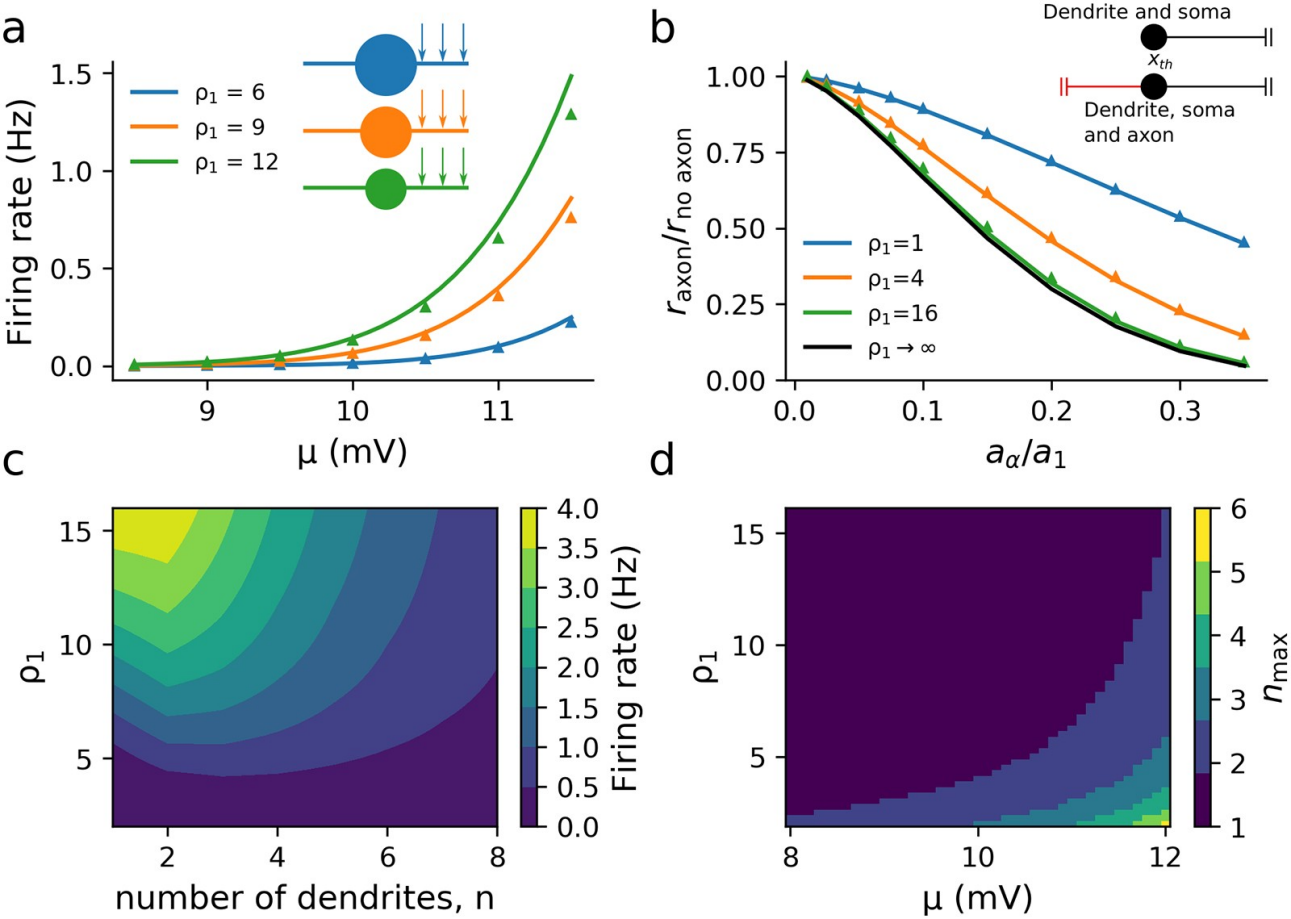
Effect of somatic impedance between a synaptically driven dendrite and axon with trigger point *x*_th_ = 30*μ*m for (a), (c) and (d), and *x*_th_ = 0*μ*m for (b). (a) The soma is characterized by the dendritic dominance factor *ρ*_1_ (see main text) with large *ρ*_1_ corresponding to a small somatic conductance. (b) A larger soma masks the effect of the axonal load on the firing rate, although this masking is negligible for smaller somata, *ρ*_1_ = 16. Fig 2a (black line) result is plotted for comparison. (c) Larger somata also reduce the firing rate in the case of *n* dendrites (*μ* = 12mV). (d) With larger *μ*, smaller *ρ*_1_ (a larger soma) increases the number of dendrites for which the firing rate is maximal, *n*_max_. Other parameters: *τ*_1_ = 10ms, *τ*_*α*_ = *τ*_0_ = 11.3–12.1ms (calculated from Eq S39), λ_1_ = 200*μ*m (kept constant with *μ* by adjusting *a*_1_, Eq S42), λ_*α*_ = 100*μ*m, *σ*_*s*_ = 3mV, *v*_th_ = 10mV, *ρ*_*α*_ calculated from Eqs (S45) or (S46).

Thus for large *n* we should expect the variance in the axon to scale as 1/*n* as before, but for smaller *n* the somatic admittance $G_{0}\gamma_{0}^{2}$ gives some key differences. We repeated the simulations for the axon-dendrite model (Fig 6), first with a single dendrite and an electrically significant soma by varying *ρ*_1_, noting that with known λ_1_ and λ_*α*_, this also determines *ρ*_*α*_, Eqs (S45, S46). Since the soma adds a conductance load *G*_0_ to the cell the overall input resistance decreases. From Eq (34), we see that this will reduce $|{\tilde{f}}_{n0}\left(\omega \right)|$ for any number of dendrites which will lower both the mean and the variance. Fig 7a shows that the effect of a larger soma (lower *ρ*_1_) lowers the firing rate.

Next, we calculated the effect of axonal load on the firing rate when we have an electrically significant soma, extending the results for the case of a nominal soma (Fig 5a). As with the nominal soma case before, we calculated the firing rate at *x*_th_ = 0 with an axon and electrically significant soma, *r*_axon_, and the firing rate of a dendrite with the same size soma without an axon, *r*_no axon_ (Fig 1a(vi)). For each somatic size, we adjusted *σ*_*s*_ so that the firing rate for a negligible axon, *a*_*α*_/*a*_1_ = 0, was fixed at 1Hz. This was done to account for the soma’s effect on the firing rate we observed earlier and we are thus solely focusing on the effect of the axonal admittance load. As we increase *a*_*α*_/*a*_1_ from zero, Fig 7b shows that *r*_axon_/*r*_no axon_ decreases more rapidly with increasing *a*_*α*_/*a*_1_ for larger *ρ*_1_ (smaller soma). This means that, in comparison to Fig 5a, the axonal load had a lower relative effect on the firing rate in the presence of a soma. This is in line with what we should expect by looking at ${\tilde{f}}_{n0}$; lower *ρ*_1_ increases the relative magnitude of $G_{0}\gamma_{0}^{2}$ in the denominator of ${\tilde{f}}_{n0}$ as compared with the axonal admittance term of $G_{\lambda_{\alpha }}\gamma_{\alpha }$.

Finally, we looked at how an electrically significant soma affects the dependence of the firing rate on the number of dendrites. By varying *ρ*_1_ and the number of dendrites *n*, Fig 7c shows that the non-monotonic dependence of the firing rate on dendritic number *n* is robust in the presence of a soma. Fig 7d illustrates that the number of dendrites that maximizes the firing rate is greater for lower *ρ*_1_ and higher *μ*. We have discussed previously why the value of *n* that maximizes firing increases with *μ* as the increase in mean from additional dendrites becomes more significant for the firing rate. Decreasing *ρ*_1_ increases the value of *n* that maximizes firing because the relative increase in conductance by adding another dendrite is smaller when the fixed somatic conductance is larger.

## Discussion

This study demonstrated how the spatio-temporal fluctuation-driven firing of neurons with dendrites, soma and axon can be approximated using the upcrossing method of Rice [27]. Despite being reduced models of neuronal structures, they provide an analytical description of a rich range of behaviours. For the one and two-dendrite models, the firing rate was shown to be independent of the electrotonic length constant; given that the length constant sets the range over which synaptic drive contributes to voltage fluctuations, this result is surprising. However, a dimensional argument extends this independence to any model in which semi-infinite neurites are joined at a point and share the same λ (any other properties without dimensions of length can be different in each neurite). The level-crossing approach provided a good approximation for the firing rate for these simple dendritic neuron models in the low-rate limit. Beyond this limit, simulations suggest that there is a universal functional form for the firing rate when parametrized by *σ*_*v*_ that is independent of both λ and the number of dendrites radiating from the nominal soma. This functional form, for coloured noise and in the white-noise limit, merits further mathematical analysis as it is distinct to that of the point-like integrate-and-fire model.

Extending the study to multiple dendrites, we showed that the firing rate depends non-monotonically on their number: adding more dendrites driven by fluctuating synaptic drive can, for a broad parameter range, decrease the fluctuation-driven firing rate. Dendritic structure has been previously shown to influence the firing rate for deterministic input [74, 75]. However, apart from the work of Tuckwell [30–32], analytical studies of stochastic drive in extended neuron models have largely focussed on a single dendrite with drive typically applied at a single point [36, 39] rather than distributed over the dendrite, or as a two-compartmental model [44]. This study demonstrates that in the low-rate regime, the upcrossing approximation allows for the analytical study of spatial models that need not be limited to a single dendrite nor with stochastic synaptic drive confined to a single point, but distributed as is the case *in vivo*.

Including axonal and somatic conductance loads demonstrated their significant effect on the firing rate—even relatively small axonal loads caused a marked reduction. Furthermore, the non-monotonic dependency of the firing rate on dendrite number was also shown to be affected by axonal radius and somatic size, demonstrating that the upcrossing method can be used to examine how structural differences in properties affect the firing rate of complex, composite, spatial neuron models.

An advantage of the level-crossing approach is it can be straightforwardly extended to include a variety of additional biophysical properties affecting neuronal integration of spatio-temporal synaptic drive. An example of this would be the inclusion of non-passive effects arising from voltage-gated currents such as *I*_*h*_ [76]. For many scenarios, particularly in the high-conductance state [77], the spatio-temporal response can be approximated as quasi-linear, allowing the voltage mean and variances to be calculated via Green’s functions using existing theoretical machinery, such as sum-over-trips on neurons [78–80]. The approach can also be extended to examine the dynamic firing-rate response to weakly modulated drive. This has already been done for point-neurons using the upcrossing method [29, 81, 82] and would only necessitate calculating the linear-response of voltage means and variances in the non-threshold case. However, for significant membrane non-linearities [83, 84] that are not sufficiently mitigated by the high-conductance state [77], the upcrossing framework developed here, predicated on Gaussian voltage fluctuations and linearity, will be inadequate. Non-linear dendritic properties—such as back-propagating action potentials or dendritic sodium spikes—support a broad variety of additional computational functions that cannot be captured by passive or quasi-active models (see [85] for a case in point). Development of a quantitative framework that includes these non-linear properties will be challenging; however, it is hoped that the linear regime considered here will provide a foundation for further work towards that end.

It can be noted that the mathematical constructions used here for the inclusion of space within neuronal structure share similarities to the framework developed for the stochastic neural field [86, 87] that models the spread of activity at the tissue scale. In the context of the neocortex, the spatial voltage variability along the principal apical dendrites of pyramidal cells would be normal to the activity spreading throughout the transverse cortical sheet. A hybrid theory might be considered which includes both these spatial mechanisms and would be an interesting topic for further study.

In summary, the extension of the upcrossing approach to spatially structured neuron models provides an analytical in-road for future studies of the firing properties of extended neuron models driven by spatio-temporal stochastic synaptic drive.

## Supporting information

S1 Methods Mathematical methods deriving the Green’s functions for each model and how the variances are calculated from them. Simulations of the upcrossing approximation’s accuracy are also described, with the results shown in Fig A.
(PDF)

S1 Code A zip file containing code in the Julia programming language that performs all of the simulations and calculations of Figs 1-7 and Fig A. Simulation and plotting of each figure is given by the file name corresponding to the figure.
(ZIP)
