# Supplementary material for: Low-rate firing limit for neurons with axon, soma and dendrites driven by spatially distributed stochastic synapses
Source: PLoS Comput Biol. 2020 Apr 20;16(4):e1007175. doi: 10.1371/journal.pcbi.1007175 (PMC7217482; doi:10.1371/journal.pcbi.1007175)
Supplement: S1 Methods — Simulations of the upcrossing approximation’s accuracy are also described, with the results shown in Fig A. (PDF) [file pcbi.1007175.s001.pdf]

Low-rate firing limit for neurons with axon, soma and dendrites driven by spatially distributed stochastic synapses Supporting Information 1 - Methods

Robert P. Gowers[1,2*], Yulia Timofeeva[3,4*], Magnus J. E. Richardson[5*]

[1]Mathematics for Real-World Systems Centre for Doctoral Training
University of Warwick, Coventry, CV4 7AL, United Kingdom,
[2] Institute for Theoretical Biology, Department of Biology, Humboldt-Universität zu Berlin,
Philippstrasse 13, Haus 4, 10115 Berlin, Germany
[3]Department of Computer Science, University of Warwick, Coventry, CV4 7AL, United Kingdom
[4] Department of Clinical and Experimental Epilepsy, UCL Queen Square Institute of Neurology,
University College London, WC1N 3BG London, United Kingdom
[5]Warwick Mathematics Institute, University of Warwick, Coventry, CV4 7AL, United Kingdom

May 1, 2020

Green's functions

In the Fourier domain, the Green's function $\tilde{\mathcal{G}}_{jk}(x_j, y_k; \omega)$ denotes the response at x_j in neurite j to an input in neurite k at y_k. For models which consist only of a single neurite, such as the infinite dendrite, these subscripts are omitted. With an additive input on neurite k in the Fourier domain $\tilde{I}(y_k; \omega)$, the voltage in neurite j in terms of frequency is given by

$$\tilde{v}_j(x_j, \omega) = \int_{\mathcal{R}} \tilde{\mathcal{G}}_{jk}(x_j, y_k; \omega)\tilde{I}(y_k; \omega)\mathrm{d}y_k, \tag{S1}$$

where $\mathcal{R}$ denotes the extent of the input neurite. After taking the inverse Fourier transform we find the voltage in the time domain in terms of a double integral

$$v_j(x_j, t) = \frac{1}{2\pi}\int_{-\infty}^{\infty} e^{i\omega t}\mathrm{d}\omega \int_{\mathcal{R}} \tilde{\mathcal{G}}_{jk}(x_j, y_k; \omega)\tilde{I}(y_k; \omega)\mathrm{d}y_k. \tag{S2}$$

The Green's function in the Fourier domain for Eq (19) satisfies the equation

$$\gamma_j^2\tilde{\mathcal{G}}_{jk} = \lambda_j^2\frac{\partial^2\tilde{\mathcal{G}}_{jk}}{\partial x_j^2} + \delta_{jk}\delta(x_j - y_k), \quad \gamma_j = \sqrt{1 + i\omega\tau_j}, \tag{S3}$$

where δ_{jk} is the Kronecker delta function. Eq (S3) has the general solution

$$\tilde{\mathcal{G}}_{jk}(x_j, y_k; \omega) = c_1 e^{-x_j\gamma_j/\lambda_j} + c_2 e^{x_j\gamma_j/\lambda_j} - \delta_{jk}\frac{\theta(x_j - y_k)}{2\gamma_j\lambda_j}[e^{(x_j-y_k)\gamma_j/\lambda_j} - e^{-(x_j-y_k)\gamma_j/\lambda_j}], \tag{S4}$$

*Correspondence: r.gowers@warwick.ac.uk, gowersro@hu-berlin.de, y.timofeeva@warwick.ac.uk, magnus.richardson@warwick.ac.uk

where the Heaviside step function $\theta(.)$ is only relevant if the input neurite and output neurite are the same. As the Green's function inherits the boundary conditions of the system it describes, we apply a model's boundary conditions to Eq (S4) to obtain the specific solution. Green's functions for each of the cases studied in this paper are given later in this document. For an infinite dendrite, the Green's function has the well-known form [1]

$$\tilde{\mathcal{G}}_\infty(x, y; \omega) = \frac{e^{-|x-y|\gamma/\lambda}}{2\gamma\lambda}. \tag{S5}$$

For multiple neurites and a soma, one can build more complex Green's functions from a generalization of Eq (S5) using the sum-over-trips formalism [2, 3]

$$\tilde{\mathcal{G}}_\infty(x_j, y_k; \omega) = \begin{cases} e^{-|x_j - y_k|\gamma_k/\lambda_k}/(2\gamma_k\lambda_k), & j = k \\ e^{-(x_j\gamma_j/\lambda_j + y_k\gamma_k/\lambda_k)}/(2\gamma_k\lambda_k), & j \neq k \end{cases}. \tag{S6}$$

The only additional calculation that needs to be made is the segment factor $\tilde{f}(\omega)$. This quantity is the ratio of the admittance of the input neurite Y_k (with $k = \alpha$ indicating the axon), to the total admittance of all neurites which radiate from the same node. For n dendrites radiating from a node with a soma this is

$$\tilde{f}(\omega) = \frac{Y_k}{Y_\alpha + Y_0 + \sum_{j=1}^{n} Y_j}. \tag{S7}$$

For each neurite, the admittance is given by

$$Y_j = 2\pi g_j a_j \lambda_j \gamma_j, \tag{S8}$$

where g_j is the total membrane conductance (including tonic synaptic conductance) while for a soma with membrane conductance G_0, the admittance is

$$Y_0 = G_0\gamma_0^2, \quad \gamma_0^2 = 1 + i\omega\tau_0. \tag{S9}$$

If there is only a single path from x_j to y_k and $j \neq k$, then the Green's function is given by Eq (S25)

$$\tilde{\mathcal{G}}_{jk}(x_j, y_k; \omega) = 2\tilde{f}(\omega)\tilde{\mathcal{G}}_\infty(x_j, y_k; \omega). \tag{S10}$$

Calculation of mean and variances

The input I to each neurite has a deterministic and stochastic component, which in the Fourier domain are

$$\tilde{I}_k = 2\pi\delta(\omega)\mu_k + \tilde{s}_k, \quad \tilde{s}_k = \frac{2\sigma_s\sqrt{\lambda_k\tau_s}\tilde{\xi}(x_k;\omega)}{1 + i\omega\tau_s}. \tag{S11}$$

Since the system is linear, this means that the voltage will have a mean and fluctuating component

$$v(x, t) = \langle v(x)\rangle + v_F(x, t). \tag{S12}$$

Substituting $\tilde{I}$ into Eq (S2) and taking the expectation, the mean in neurite j due to input in k is

$$\langle v_{jk}(x_j)\rangle = \mu_k \int_{\mathcal{R}} \tilde{\mathcal{G}}_{jk}(x_j, y_k; 0)\mathrm{d}y_k \tag{S13}$$

while the fluctuating component is

$$v_{Fjk}(x_j, t) = \frac{\sigma_s\sqrt{\lambda_k\tau_s}}{\pi} \int_{-\infty}^{\infty} e^{i\omega t}\mathrm{d}\omega \int_{\mathcal{R}} \frac{\tilde{\mathcal{G}}_{jk}(x_j, y_k; \omega)}{1 + i\omega\tau_s}\tilde{\xi}(y_k;\omega)\mathrm{d}y_k. \tag{S14}$$

Thus the variance contribution from neurite k is obtained by squaring v_{Fjk} and taking the expectation, noting that $\langle\tilde{\xi}_j(y_j,\omega)\tilde{\xi}_k^*(y_k',\omega')\rangle = 2\pi\delta_{jk}\delta(y-y')\delta(\omega-\omega')$ [1]

$$\sigma_{vjk}^2(x_j) = \frac{2\sigma_s^2\lambda_k\tau_s}{\pi}\int_{-\infty}^{\infty}\mathrm{d}\omega\int_{\mathcal{R}}\frac{|\tilde{\mathcal{G}}_{jk}(x_j,y_k;\omega)|^2}{1+\omega^2\tau_s^2}\mathrm{d}y_k, \tag{S15}$$

and similarly the variance of the voltage time derivative is found by multiplying the integrand of Eq (S15) by ω^2

$$\sigma_{\dot{v}jk}^2(x_j) = \frac{2\sigma_s^2\lambda_k\tau_s}{\pi}\int_{-\infty}^{\infty}\mathrm{d}\omega\int_{\mathcal{R}}\frac{\omega^2|\tilde{\mathcal{G}}_{jk}(x_j,y_k;\omega)|^2}{1+\omega^2\tau_s^2}\mathrm{d}y_k. \tag{S16}$$

This approach is equivalent to that found in [4], where the integrand of Eqs (S15, S16) is proportional to the power spectral density of the voltage.

For n dendrites with synaptic input, the response in the axon is simply the linear sum from each dendrite,

$$\langle v_\alpha(x_\alpha)\rangle = \sum_{k=1}^{n}\langle v_{\alpha k}(x_\alpha)\rangle, \quad v_{F\alpha}(x_\alpha) = \sum_{k=1}^{n}v_{F\alpha k}(x_\alpha), \tag{S17}$$

and since the stochastic drive between dendrites is uncorrelated, the variance contributions from each dendrite also sum linearly,

$$\sigma_v^2(x_\alpha) = \sum_{k=1}^{n}\sigma_{v_\alpha k}^2(x_\alpha), \quad \sigma_{\dot{v}}^2(x_\alpha) = \sum_{k=1}^{n}\sigma_{\dot{v}_\alpha k}^2(x_\alpha). \tag{S18}$$

For dendrites with identical properties and drive, this means we can multiply Eqs (S15, S16) by n to obtain the total values of σ_v^2 and $\sigma_{\dot{v}}^2$.

In all the cases given, $\langle v\rangle$ is analytically calculable. For the infinite dendrite $\langle v\rangle = \mu$, while the resting potential in the axon for n dendrites is

$$\langle v(x_\alpha)\rangle = n\mu e^{-x_\alpha/\lambda_\alpha}\tilde{f}_n(0) \tag{S19}$$

and the addition of a soma changes this to

$$\langle v(x_\alpha)\rangle = n\mu e^{-x_\alpha/\lambda_\alpha}\tilde{f}_{n0}(0). \tag{S20}$$

For many simple cases - such as the sealed dendrite, one-dendrite, and two-dendrite models - closed-form expressions for the variances are attainable. For all cases with an axon and/or soma with different membrane properties to the dendrite, the ω-integral can be calculated numerically or approximated in a limit of interest. However, given the n in the denominator of $\tilde{f}_n$ and $\tilde{f}_{n0}$, we expect from Eqs (S15, S16) that the variances scale as $\sim 1/n$ for large n.

Derivation of Green's functions

Closed dendrite

For the closed dendrite with length L, the region of integration is $\mathcal{R} = [0, L]$. Given the zero-current boundary conditions at the ends $(x = 0,\ x = L)$

$$\left.\frac{\partial v}{\partial x}\right|_{x=0} = 0 = \left.\frac{\partial v}{\partial x}\right|_{x=L}, \tag{S21}$$

we can solve the Green's function differential equation, Eq(S3), to obtain [1]

$$\tilde{\mathcal{G}}(x,y;\omega) = \frac{\cosh[(L-|x-y|)\gamma/\lambda] + \cosh[(L-|x+y|)\gamma/\lambda]}{2\lambda\gamma\sinh(L\gamma/\lambda)}. \tag{S22}$$

[1] $\xi(y,\omega) = \int_{-\infty}^{\infty}\xi(y,t)e^{-i\omega t}\mathrm{d}t, \quad \xi^*(y',\omega') = \int_{-\infty}^{\infty}\xi(y',t')e^{i\omega' t'}\mathrm{d}t'$

$\langle\xi(y,\omega)\xi^*(y',\omega')\rangle = \int_{-\infty}^{\infty}\mathrm{d}t\int_{-\infty}^{\infty}\langle\xi(y,t)\xi(y',t')\rangle e^{-i\omega t}e^{i\omega' t'}\mathrm{d}t' = \delta(y-y')\int_{-\infty}^{\infty}e^{-i(\omega-\omega')t}\mathrm{d}t$

$\therefore \langle\xi(y,\omega)\xi^*(y',\omega')\rangle = 2\pi\delta(y-y')\delta(\omega-\omega')$.

Dendrite and axon

Using the sum-over-trips method, $\tilde{\mathcal{G}}_{jk}$ is given by the sum of infinite Green's functions of each path which traces back from output position x_j to input position y_k. If a given path has length l_{trip}, then we represent this sum as [2, 3]

$$\tilde{\mathcal{G}}_{jk}(x_j, y_k; \omega) = \sum_{\text{trips}} A_{\text{trip}}(\omega) \tilde{\mathcal{G}}_{\infty}(l_{\text{trip}}; \omega), \tag{S23}$$

where A_{trip} is the trip coefficient that depends on the intersections between cables that a trip must path through. Since the neurites we consider are semi-infinite, there is only a single trip for a path from the axon to the input dendrite (however, the sum-over-trips approach provides a method for straightforward generalization to dendrites with closed ends). The only trip coefficient required is that for transmission through a node which is given by $A_{\text{trip}} = 2\tilde{f}(\omega)$ [3]. Therefore, the Green's function from the dendrite $k = 1$ to the axon, $j = \alpha$, is given by

$$\tilde{\mathcal{G}}_{\alpha 1}(x_\alpha, y_1; \omega) = 2\tilde{f}(\omega) \tilde{\mathcal{G}}_{\infty}(x_\alpha, y_1; \omega), \tag{S24}$$

which upon substitution of $\tilde{f}$ and $\tilde{\mathcal{G}}_{\infty}$ yields

$$\tilde{\mathcal{G}}_{\alpha 1}(x_\alpha, y_1; \omega) = \frac{g_1^2 \lambda_1^3 \gamma_1}{\lambda_1 \gamma_1 (g_1^2 \lambda_1^3 \gamma_1 + g_\alpha^2 \lambda_\alpha^3 \gamma_\alpha)} e^{-(x_\alpha \gamma_\alpha / \lambda_\alpha + y_1 \gamma_1 / \lambda_1)}. \tag{S25}$$

Multiple dendrites and axon

When there are n dendrites, the segment factor becomes

$$\tilde{f}_n(\omega) = \frac{g_1^2 \lambda_1^3 \gamma_1}{n g_1^2 \lambda_1^3 \gamma_1 + g_\alpha^2 \lambda_\alpha^3 \gamma_\alpha}, \tag{S26}$$

and hence the Green's function for the axonal response is

$$\tilde{\mathcal{G}}_{\alpha 1}(x_\alpha, y_1; \omega) = \frac{g_1^2 \lambda_1^3 \gamma_1}{\lambda_1 \gamma_1 (n g_1^2 \lambda_1^3 \gamma_1 + g_\alpha^2 \lambda_\alpha^3 \gamma_\alpha)} e^{-(x_\alpha \gamma_\alpha / \lambda_\alpha + y_1 \gamma_1 / \lambda_1)}. \tag{S27}$$

Since all dendrites have the same properties for this model, we can then claim that $\tilde{\mathcal{G}}_{\alpha 1} = \tilde{\mathcal{G}}_{\alpha 2} = ... = \tilde{\mathcal{G}}_{\alpha n}$.

Dendrites, soma and axon

For an electrically significant soma, the segment factor is now

$$\tilde{f}_{n0}(\omega) = \frac{\rho_1 \gamma_1}{\gamma_0^2 + n \rho_1 \gamma_1 + \rho_\alpha \gamma_\alpha}, \tag{S28}$$

hence the Green's function is

$$\tilde{\mathcal{G}}_{\alpha 1}(x_\alpha, y_1; \omega) = \frac{\rho_1 \gamma_1}{\lambda_1 \gamma_1 (\gamma_0^2 + n \rho_1 \gamma_1 + \rho_\alpha \gamma_\alpha)} e^{-(x_\alpha \gamma_\alpha / \lambda_\alpha + y_1 \gamma_1 / \lambda_1)}. \tag{S29}$$

Derivation of variances

While there are other methods for obtaining variances that may be more convenient for the models which provide closed-form solutions (such as a Green's functions in time [5] or Fourier series decomposition [5, 6, 7]) the method we present here extends most easily to arbitrary neuronal structures. For clarity of explanation, we derive the two-dendrite model first.

Two-dendrite model

For the two-dendrite model $|\tilde{\mathcal{G}}|^2$ is given by

$$|\tilde{\mathcal{G}}(x,y;\omega)|^2 = \frac{e^{-|x-y|z/\lambda}}{4\lambda^2|\gamma|^2}, \tag{S30}$$

where the definition $z_j = \gamma_j + \gamma_j^*$ is useful for keeping the algebra compact. We can readily integrate $|\tilde{\mathcal{G}}|^2$ with respect to y after substituting into Eq (S15) with $\mathcal{R} = (-\infty, \infty)$ to obtain a result in which all factors of λ cancel

$$\sigma_v^2 = \frac{\sigma_s^2 \tau_s}{\pi} \int_{-\infty}^{\infty} \frac{\mathrm{d}\omega}{z|\gamma|^2(1+\omega^2\tau_s^2)}. \tag{S31}$$

Using the substitution $\omega\tau_v = 2q\sqrt{q^2+1}$ and splitting into partial fractions this integral becomes

$$\sigma_v^2 = \frac{2\sigma_s^2\tau_s}{\pi\tau_v} \left\{ \int_0^{\infty} \frac{\mathrm{d}q}{q^2+1} - \int_0^{\infty} \frac{4\tau_s^2 q^2 \mathrm{d}q}{\tau_v^2 + 4\tau_s^2 q^2(q^2+1)} \right\}, \tag{S32}$$

which can be resolved to give Eq (26).

One-dendrite model

Defining $iu = \gamma - \gamma^*$ we find

$$|\tilde{\mathcal{G}}(x,y;\omega)|^2 = \begin{cases} e^{-xz/\lambda}(e^{yz/\lambda} + e^{-yz/\lambda} + e^{iyu/\lambda} + e^{-iyu/\lambda})/(4\lambda^2|\gamma|^2), & y < x \\ e^{-yz/\lambda}(e^{xz/\lambda} + e^{-xz/\lambda} + e^{ixu/\lambda} + e^{-ixu/\lambda})/(4\lambda^2|\gamma|^2), & y > x \end{cases}, \tag{S33}$$

which integrates with respect to y with $\mathcal{R} \in [0, \infty)$, giving

$$\sigma_v^2 = \frac{\sigma_s^2\tau_s}{\pi} \int_{-\infty}^{\infty} \frac{\mathrm{d}\omega}{|\gamma|^2(1+\omega^2\tau_s^2)} \left[\frac{1}{z} + e^{-xz/\lambda}\frac{\sin(xu/\lambda)}{u} + e^{-xz/\lambda}\frac{\cos(xu/\lambda)}{z} \right]. \tag{S34}$$

We can see that at $x = 0$ the variance is double the two-dendrite case and as $x \to \infty$ the variance becomes equal to the two-dendrite case. For general x we can change the integration variable in a similar manner to the two-dendrite model to obtain the desired result.

Closed dendrite

With the closed dendrite, $|\tilde{\mathcal{G}}|^2$ is more lengthy

$$\begin{aligned} |\tilde{\mathcal{G}}(x,y;\omega)|^2 = & \left\{ \tfrac{1}{2}\cosh[(L-x+y)z/\lambda] + \tfrac{1}{2}\cos[(L-x+y)u/\lambda] \right. \\ & + \tfrac{1}{2}\cosh[(L-x-y)z/\lambda] + \tfrac{1}{2}\cos[(L-x-y)u/\lambda] \\ & \left. + \cosh[(L-x)z/\lambda]\cos(yu/\lambda) + \cosh(yz/\lambda)\cos[(L-x)u/\lambda] \right\}/D, \quad y < x \qquad \text{(S35)} \\ = & \left\{ \tfrac{1}{2}\cosh[(L+x-y)z/\lambda] + \tfrac{1}{2}\cos[(L+x-y)u/\lambda] \right. \\ & + \tfrac{1}{2}\cosh[(L-x-y)z/\lambda] + \tfrac{1}{2}\cos[(L-x-y)u/\lambda] \\ & \left. + \cosh[(L-y)z/\lambda]\cos(xu/\lambda) + \cosh(xz/\lambda)\cos[(L-y)u/\lambda] \right\}/D, \quad y > x \qquad \text{(S36)} \end{aligned}$$

where $D = 2\lambda^2|\gamma|^2[\cosh(Lz/\lambda) - \cos(Lu/\lambda)]$; however, we can see that all the functions involved will have closed-form integrals with respect to y.

Dendrite and axon

For the dendrite and axon, we will leave $|\tilde{\mathcal{G}}|^2$ in terms of the segment factor to show how this approach extends to multiple dendrites and the addition of a soma

$$|\tilde{\mathcal{G}}_{\alpha 1}(x_\alpha, y_1; \omega)|^2 = \frac{|\tilde{f}_1(\omega)|^2}{\lambda_1^2 |\gamma_1|^2} e^{-(x_\alpha z_\alpha/\lambda_\alpha + y_1 z_1/\lambda_1)}. \tag{S37}$$

Integrating with respect to y with $\mathcal{R} = [0, \infty)$ gives,

$$\sigma_v^2(x_\alpha) = \frac{2\sigma_s^2 \tau_s}{\pi} \int_{-\infty}^{\infty} \frac{|\tilde{f}_1(\omega)|^2 e^{-x_\alpha z_\alpha/\lambda_\alpha}}{z_1 |\gamma_1|^2 (1 + \omega^2 \tau_s^2)} \mathrm{d}\omega, \tag{S38}$$

which generalizes to n dendrites or the addition of a soma by replacing $|\tilde{f}_1|^2$ with $|\tilde{f}_n|^2$ or $|\tilde{f}_{n0}(\omega)|^2$ respectively.

Calculation of axonal parameters

Assuming that the following parameters have the same value in the dendrite and axon: E_L, g_L and c_m, we can express the axonal parameters in terms of the dendritic ones. Since there is no synaptic drive in the axon, $g_\alpha = g_L$, while in the dendrite $g_1 = g_L + \langle g_s \rangle$. We denote the ratio between the membrane time constants as ϵ, which given constant c_m is

$$\epsilon = \frac{\tau_\alpha}{\tau_1} = \frac{g_1}{g_\alpha} = \frac{g_L + \langle g_s \rangle}{g_L}. \tag{S39}$$

Recalling our definitions of E and μ in the dendrite as,

$$E = \frac{E_L g_L + E_s \langle g_s \rangle}{g_L + \langle g_s \rangle}, \quad \mu = E - E_L, \tag{S40}$$

we can rearrange to find an expression for ϵ in terms of potentials alone,

$$\epsilon = \frac{E_L - E_s}{E_L + \mu - E_s}. \tag{S41}$$

Hence we can calculate τ_α in terms of τ_1 given μ, E_L and E_s. For $E_L = -70\mathrm{mV}$, $E_s = 0\mathrm{mV}$ and $\mu = 10\mathrm{mV}$ this results in $\epsilon = 7/6$.

When a_α and a_1 are fixed - as in Figs 5, 6c, and 7b - we can calculated λ_α in terms of a given λ_1. Recalling the definition of the length constant from Eq (7) and making the reasonable assumption that the axial resistivity r_a is the same in the dendrite and axon, we have

$$\lambda_1 = \sqrt{\frac{a_1}{2 g_1 r_\mathrm{a}}}, \quad \lambda_\alpha = \sqrt{\frac{a_\alpha}{2 g_\alpha r_\mathrm{a}}}, \quad \frac{\lambda_\alpha}{\lambda_1} = \sqrt{\frac{a_\alpha g_1}{a_1 g_\alpha}}. \tag{S42}$$

Taking $g_\alpha = g_L$ again and our earlier definition of ϵ in Eq (S39), we can write this as

$$\frac{\lambda_\alpha}{\lambda_1} = \sqrt{\epsilon \frac{a_\alpha}{a_1}}. \tag{S43}$$

Finally, for the electrically significant soma in Fig 7 we give ρ_1 but not ρ_α, noting that it can be calculated given λ_1 and λ_α or a_1 and a_α. Recalling our definitions of ρ and ϵ we find

$$\rho_1 = \frac{2\pi a_1 g_1 \lambda_1}{G_0}, \quad \rho_\alpha = \frac{2\pi a_\alpha g_\alpha \lambda_\alpha}{G_0}, \quad \frac{\rho_\alpha}{\rho_1} = \frac{1}{\epsilon} \frac{a_\alpha \lambda_\alpha}{a_1 \lambda_1}. \tag{S44}$$

Hence when λ_1 and λ_α are fixed as in Fig 7a,c,d we have

$$\frac{\rho_\alpha}{\rho_1} = \frac{1}{\epsilon^2} \frac{\lambda_\alpha^3}{\lambda_1^3}, \tag{S45}$$

while when a_1 and a_α are fixed as in Fig 7b

$$\frac{\rho_\alpha}{\rho_1} = \frac{1}{\sqrt{\epsilon}} \frac{a_\alpha^{3/2}}{a_1^{3/2}}. \tag{S46}$$

Accuracy of the upcrossing approximation

The accuracy of the upcrossing approximation in the one-dendrite model was assessed using the spike-coincidence measure Γ [8] between two simulated processes subject to the same synaptic drive, one with reset and one without (as shown earlier by the orange and blue traces in Fig 1b). This measure was chosen because it highlights not only if the mean upcrossing rate is similar to the threshold-reset rate, but also whether the upcrossing events occur at similar times to the reset events. For a time window Δ over which to determine coincident events

$$\Gamma = \frac{2(N_c - \langle N_c \rangle)}{n(N_{\mathrm{tr}} + N_{\mathrm{uc}})}, \quad n = 1 - 2r_{\mathrm{uc}}\Delta, \tag{S47}$$

where N_c is the number of simulated coincident events, N_{tr} is the number of threshold-reset events, N_{uc} is the number of upcrossing events, and $\langle N_c \rangle = 2r_{uc}N_{\mathrm{tr}}\Delta$ is the expected number of coincident events between the threshold-reset process and a Poisson process with rate r_{uc}.

Letting the time window be approximately similar to the AP width, $\Delta = 2$ms, we performed simulations in terms of two dimensionless parameters: the distance between the threshold and the mean in terms of the voltage standard deviation, $(v_{\mathrm{th}} - \langle v \rangle)/\sigma_v$, and the relative synaptic timescale τ_s/τ_v. Fig A shows that the coincidence factor increases when fluctuations reach threshold more rarely (higher $(v_{\mathrm{th}} - \langle v \rangle)/\sigma_v$). This is in line with what we would expect; as threshold-crossing events become rarer, there is more time between events for the upcrossing and reset voltage traces to reconverge.

We also found that Γ peaks for intermediate τ_s/τ_v, implying that the upcrossing approximation is worse for both rapidly filtered noise ($\tau_s \to 0$) and slowly filtered noise ($\tau_s \to \infty$). When τ_s/τ_v is small, voltage fluctuations are more rapid and hence several upcrossing events may occur about threshold in quick succession with fewer corresponding threshold-reset events, decreasing Γ. Lower Γ from higher τ_v/τ_s arises from excursions above threshold after an upcrossing event. These excursions increase in duration with higher τ_s, giving a higher probability that a threshold-reset event occurs before the two voltage traces converge. These synaptic filtering effects qualitatively agree with those found for the point-neuron model in [9], for which the accuracy of the upcrossing approximation is maximized near $\tau_s/\tau_v \sim 1$.

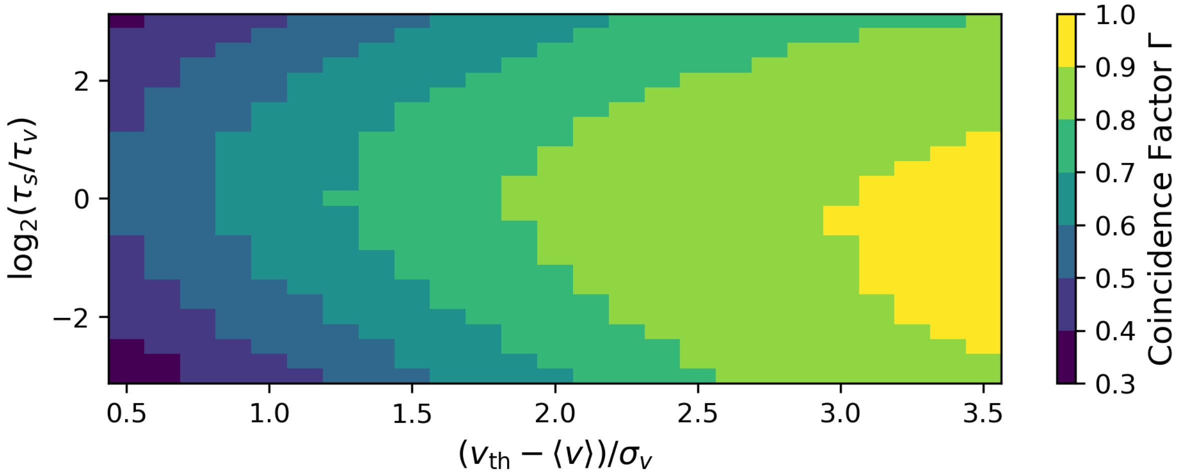

Fig A. A comparison of simulated threshold-reset and upcrossing events in the one-dendrite model shows that the coincidence factor Γ (Eq S47) increases with $(v_{\mathrm{th}} - \langle v \rangle)/\sigma_v$, but varies non-monotonically with τ_s/τ_v, peaking around $\tau_s/\tau_v \sim 0.5$. Other parameters: $\tau_v = 10$ms, $\Delta = 2$ms.

References

[1] Tuckwell HC. Time-dependent cable theory for nerve cylinders and dendritic trees. In: Introduction to theoretical neurobiology: volume 1, linear cable theory and dendritic structure. Cambridge University Press; 1988. p. 180–233.

[2] Abbott LF, Farhi E, Gutmann S. The path integral for dendritic trees. Biological Cybernetics. 1991;66(1):49–60.

[3] Coombes S, Timofeeva Y, Svensson CM, Lord GJ, Josić K, Cox SJ, et al. Branching dendrites with resonant membrane: a "sum-over-trips" approach. Biological Cybernetics. 2007;97(2):137–149. doi:10.1007/s00422-007-0161-5.

[4] Manwani A, Koch C. Detecting and Estimating Signals in Noisy Cable Structures, I: Neuronal Noise Sources. Neural Computation. 1999;11(8):1797–1829.

[5] Tuckwell HC, Walsh JB. Random currents through nerve membranes - I. Uniform poisson or white noise current in one-dimensional cables. Biol Cybern. 1983;49(2):99–110. doi:10.1007/BF00320390.

[6] Tuckwell HC. Spatial neuron model with two-parameter Ornstein-Uhlenbeck input current. Physica A: Statistical Mechanics and its Applications. 2006;368(2):495–510. doi:10.1016/j.physa.2005.12.022.

[7] Tuckwell HC. Analytical and simulation results for the stochastic spatial FitzHugh-Nagumo model neuron. Neural Computation. 2008;20(12):3003–3033.

[8] Jolivet R and Gerstner W Predicting spike times of a detailed conductance-based neuron model driven by stochastic spike arrival. Journal of Physiology-Paris 2004; 98(4-6): 442-451.

[9] Badel L. Firing statistics and correlations in spiking neurons: A level-crossing approach. Physical Review E. 2011;84(4):041919. doi:10.1103/PhysRevE.84.041919.
